# Scarf enables a highly memory-efficient analysis of large-scale single-cell genomics data

**DOI:** 10.1038/s41467-022-32097-3

**Published:** 2022-08-08

**Authors:** Parashar Dhapola, Johan Rodhe, Rasmus Olofzon, Thomas Bonald, Eva Erlandsson, Shamit Soneji, Göran Karlsson

**Affiliations:** 1 Division of Molecular Hematology, Lund Stem Cell Center, Lund University, Lund, Sweden; 2 Institut Polytechnique de Paris, Paris, France

**Keywords:** Genome informatics, Software

## Abstract

As the scale of single-cell genomics experiments grows into the millions, the computational requirements to process this data are beyond the reach of many. Herein we present Scarf, a modularly designed Python package that seamlessly interoperates with other single-cell toolkits and allows for memory-efficient single-cell analysis of millions of cells on a laptop or low-cost devices like single-board computers. We demonstrate Scarf’s memory and compute-time efficiency by applying it to the largest existing single-cell RNA-Seq and ATAC-Seq datasets. Scarf wraps memory-efficient implementations of a graph-based t-stochastic neighbour embedding and hierarchical clustering algorithm. Moreover, Scarf performs accurate reference-anchored mapping of datasets while maintaining memory efficiency. By implementing a subsampling algorithm, Scarf additionally has the capacity to generate representative sampling of cells from a given dataset wherein rare cell populations and lineage differentiation trajectories are conserved. Together, Scarf provides a framework wherein any researcher can perform advanced processing, subsampling, reanalysis, and integration of atlas-scale datasets on standard laptop computers. Scarf is available on Github: https://github.com/parashardhapola/scarf.

## Abstract (web summary)

As the scale of single-cell genomics experiments grows into the millions, the computational requirements to process this data are beyond the reach of many. Here the authors present Scarf, a modularly designed Python package that makes the analysis workflow highly memory efficient such that even the largest existing datasets can be analyzed on an average modern laptop.

## Introduction

The rapid evolution, integration, and diversification of high-throughput single-cell genomic technologies continue to have a critical impact on our conceptual understanding of tissue heterogeneity, cell-type specification, and differentiation. Accumulating technological advances reaches beyond gene expression quantification to the measurement of higher-dimensional genomic features and an exponential increase of input cell numbers^1^. Single-cell genomic data from large-scale studies are often made available through web portals where quick but limited access is granted, restricting how much information can be mined.

A major objective for computational analysis of single-cell genomic data has been to improve the scalability of analysis for increasing cell numbers and features^2^. While recent focus has been to improve computation time, memory scalability has largely been ignored even though memory capacity in computing systems is currently the major hurdle for increased usage of single-cell genomic datasets. Larger datasets with more than 100,000 cells obligate the use of specialised hardware with larger primary memory capacity (random access memory, RAM) in the order of several hundreds of gigabytes^3^. This resource barrier prevents a majority of biologists and bioinformaticians from uncomplicated access to their own as well as publicly available datasets.

We have developed Scarf, which enables analysis of even the largest single-cell datasets with a limited amount of RAM consumption. Consequently, users can now analyse atlas-scale datasets on their laptop computers and for the first time, perform large-scale multi-atlas analysis on servers. Upon the core memory efficient architecture of Scarf, we have introduced multiple novel algorithms to solve the challenges specifically posed by atlas-scale datasets. Briefly, we have developed a cell subsampling algorithm built upon Scarf that can allow generating highly representative subsets of data for usage in other tools. We have introduced a visualisation and clustering algorithm, that were previously unknown in the single-cell field, for a faster and more accurate representation of single-cell datasets.

Scarf is a modularly developed and extensible Python package, designed to work with any kind of single-cell genomic dataset presented as a matrix of cells and features. In the current version, functionality to analyse ScRNA-Seq^4^, ScATAC-Seq^5,6^ and CITE-Seq^7^ datasets are included. Scarf performs major steps of data analysis like data filtering, normalisation, feature selection, linear dimension reduction, cell-cell neighbourhood graph creation, non-linear dimension reduction, clustering and identification of discriminatory features.

For an efficient and interpretable clustering of cells at low memory consumption, we introduce Paris^8^, a hierarchical graph clustering algorithm scalable to millions of cells included in Scarf. For the visualisation of generated cell clusters, the Uniform Manifold Approximation and Projection (UMAP)^9^ algorithm is complemented with graph t-distributed stochastic neighbour embedding (t-SNE)^10^. We show when the same initialisation of data is used the UMAP will highlight the magnitude of cluster relations, while t-SNE reveals the heterogeneity within the data. Moreover, by creating a graph-based method for cell subsampling (subsampling henceforth), we have leveraged Scarf’s memory efficiency while preserving archetypes or differentiation trajectories.

Finally, Scarf is equipped with features for optimal data integration where samples are projected onto each other cell by cell, using a K-Nearest neighbour (KNN) mapping approach. This strategy avoids the generation of non-biological sample separation when datasets generated from different technologies or techniques need to be combined, or from strong molecular signals when comparing cells, for example, after perturbation experiments or malignant transformation.

Importantly, benchmarking performed by utilising the largest publicly available scRNA-Seq and scATAC-Seq datasets show that Scarf can handle millions of cells and hundreds of thousands of features on a regular laptop in a time-efficient manner while producing results that are consistent with the existing tools that are widely used but memory-exhausting.

Scarf is powerful in scenarios where researchers need to reanalyse existing atlas-scale datasets to obtain a relevant subset of cells, or for integration with their own data. With an ever-increasing growth of single-cell datasets, it is imperative that researchers can easily build upon existing datasets at any scale without being limited by computational resources.

## Results

### Scarf enables analysis of atlas-scale scRNA-Seq and scATAC-Seq datasets on laptop computers

**Fig. 1:**
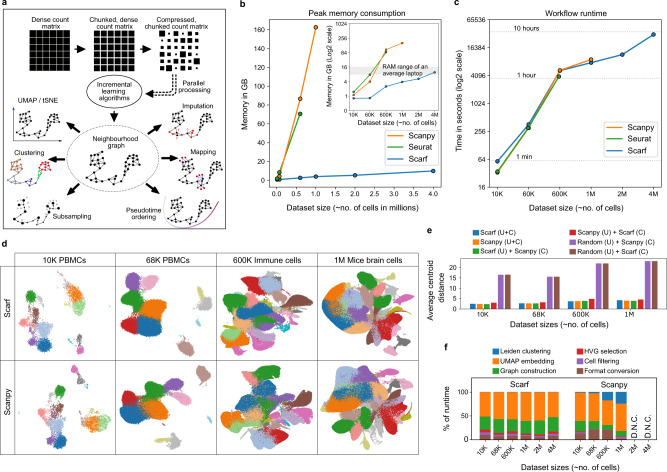
Scarf performs memory and time-efficient computation to produce consistent embedding and clustering. **a** Schematic of the workflow of Scarf wherein the input data is illustrated as a matrix used to generate a cell-cell neighbourhood graph. Outward pointing arrows from the neighbourhood graph indicate the operations that can be performed on the graph in no particular order. **b** Plot showing the amount of memory consumed by Scarf, Seurat and Scanpy on datasets containing up to approx. 4 million cells. The inset image shows the same data with the *y*-axis on the log2 scale. Dots connecting the lines indicate the number of cells on the *x*-axis and corresponding memory consumed on the y-axis. Lines are drawn to indicate a general trend. **c** Plot showing the amount of time (in seconds) consumed by Scarf, Seurat and Scanpy on the six datasets used for benchmarking. The x-axis shows the number of cells in the datasets as categorical labels. Horizontal dotted lines indicate the time consumed (in hours). **d** Plots showing UMAP embedding of cells calculated using Scarf and Scanpy. Cells are coloured, for both Scarf and Scanpy, by the cluster identity obtained using Scarf’s Leiden clustering. Only four of the six datasets, that were successfully processed using Scanpy are shown here. **e** Bar plots showing the average distance (in UMAP space) of cells from their corresponding cluster centroids. ‘U’ = UMAP and ‘C’ = clustering. **f** Percentage of time consumed by six broad steps in the processing pipeline of Scarf and Scanpy. D.N.C = ‘did not complete’ due to out-of-memory error. Please note that the Leiden clustering step might not be visible for Scarf when zoomed out because of its quick runtime.

Single-cell genomic datasets undergo two stages of processing. In the first stage, sequencing reads are used to generate a count matrix which serves as the foundation for all the downstream analyses. The two axes of the count matrix usually consist of cell barcodes and features. For example, in single-cell RNA-Seq data, features normally represent genes/transcripts and for single-cell ATAC-Seq data features are the peak coordinates. Scarf achieves memory efficiency by dividing a dataset into small chunks and then compressing these chunks individually and storing them onto the disk (Fig. 1a). Chunks with a larger number of zero values automatically achieve a larger degree of compression. In contrast to other single-cell libraries like Loompy and Scanpy^11^, Scarf, by default, uses the Zarr file format^12^ to store chunked datasets and not HDF5^13^. Zarr improves support and performance for parallel operations as well as interactions with datasets that are stored remotely. Algorithms like PCA (principal component analysis) and KNN (k-nearest neighbours), in most commonly used implementations, require the whole dataset to be loaded in memory as input. Scarf uses out-of-core (a.k.a. incremental) implementations of these algorithms that allow the iterative input of data in small chunks. These incremental learning algorithms lead to the creation of a cell-cell neighbourhood graph structure of the data (see Methods for details) which can subsequently be used for downstream steps like generating UMAP/t-SNE visualisations, pseudotime ordering, etc.

One of the most common objectives of a single-cell analysis pipeline is to generate a low-dimensional embedding of cells (e.g. UMAP and t-SNE) and to partition them into distinct clusters. Along with UMAP/t-SNE embedding and clustering, current protocols involve common processes like data normalisation, feature selection, dimension reduction and cell neighbourhood graph generation, forming a basic workflow of single-cell data^14^. We benchmarked the time and memory consumption of such a basic workflow using Scarf and two other widely used scRNA-Seq analysis toolkits Scanpy^11^ and Seurat^15^.

Using six scRNA-Seq datasets^16–18^ with increasing cell numbers, we found that under the same analysis parameters (see Methods), Scarf had substantially lower memory consumption than Scanpy and Seurat (Fig. 1b). Importantly, using less than 16 GB of memory (RAM) (commonly available in modern laptops), even the largest set of four million cells^18^ were efficiently processed by Scarf. In contrast, Scanpy was not able to process the two and four million cell datasets due to high memory consumption, despite 200 GB of RAM being available during the benchmarking experiments. Seurat was not able to handle the loading of the 1M cell dataset or larger due to limitations of matrix sizes in R language. For the datasets that both Seurat and Scanpy were able to process, the memory consumption was similar and many folds higher than Scarf. For the largest set successfully analysed by Scanpy (one million brain cell dataset generated by 10x genomics), ~40 times more RAM was used compared to Scarf, under the same parameters and equivalent steps. Moreover, the lower memory consumption by Scarf did not come at a cost of the runtime of the workflow, which was similar across all three datasets (Fig. 1c). Of note, Scarf was able the process all analysis steps using the four million cell dataset within 10 h without exceeding 16GB of memory usage.

The four datasets that could be processed by Scanpy, were visualised by the UMAP embeddings generated by either Scarf or Scanpy (Fig. 1d). To aid the visual inspection, cells were coloured based on their cluster identity obtained using Scarf. The generated UMAPs indicate that very similar embeddings of cells were achieved. To quantify any potential differences, we calculated the average centroid distance (ACD), i.e. the average Euclidean distance of cells from their cluster centroids in the UMAP space and cross-evaluation was performed using UMAP from one and clustering information from the other pipeline. Indeed, ACD values were found to be similar when comparing the two pipelines to the ones obtained when both UMAP and clustering information was obtained from the same pipeline (Fig. 1e). In contrast, ACD values were substantially higher after generating a random embedding of cells.

Next, extensive benchmarking was conducted under different combinations of three variable parameters: number of highly variable genes (HVGs), number of PCA components and neighbours to be used in the construction of the cell-cell neighbourhood graph. We found that memory consumption was consistently and substantially lower in Scarf than Scanpy, across all the parameters tested (Supplementary Figs. 1 and 2). The number of HVGs and PCA dimensions used had a very low impact on memory consumption and runtime. However, using larger numbers of neighbours for graph building (parameter ‘k’) led to a substantial increment in memory consumption. We investigated the runtime of each major stage of the pipeline and found that the proportion of time consumed by each stage was similar across the benchmarked datasets (Fig. 1f). With the chosen parameters, UMAP was the longest-running step for both Scanpy and Scarf, occupying more than 50% of the runtime. In the case of Scanpy, the fraction of time taken by Leiden clustering increased with larger data sizes (from), while in the case of Scarf, it remained consistently low across datasets.

**Fig. 2:**
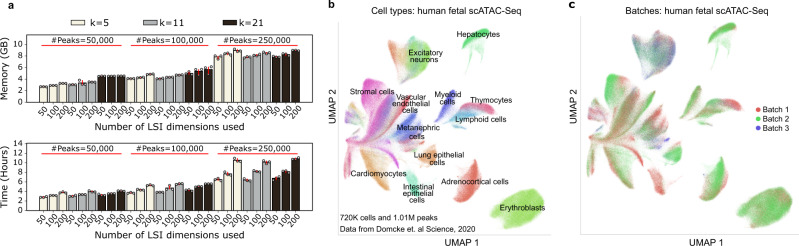
Scarf enables memory-efficient analysis of large-scale scATAC-Seq datasets. **a** Bar plots showing the memory and time consumption of Scarf on the >700 K single-cell ATAC-Seq dataset. Each bar (mean value of three technical runs) shows the result of benchmarking conducted using a combination of multiple numbers of peaks, *k* (number of neighbours in the nearest neighbour graph) and number of latent semantic indexing dimensions used over which the graph was computed. The error bars show the standard deviation computed over three iterations of the entire pipeline (individual datapoints shown as empty circles). **b** Scatter plot showing UMAP embedding of the cells from the >700 K single-cell ATAC-Seq dataset. Cells are coloured by author-annotated cell types or (**c)** batches of data generation.

Scarf can also be applied to other single-cell genomics methods, including large-scale scATAC-Seq. To further demonstrate Scarf’s scalability, we used a dataset generated from human foetal samples representing 15 organs, 720,613 cells in total and a feature set comprising 1.05 million peak regions^19^. Scarf was able to process this data, generating clusters and UMAP embeddings, in ~5.5 h using less than 2.5 GB of RAM. As done previously for the scRNA-Seq datasets, we benchmarked the runtime and memory consumption across three critical parameters: the number of peaks, latent semantic indexing (LSI) components used, and the number of neighbours identified for neighbourhood graph creation (Fig. 2a). Due to the large size of the peak set present, unlike scRNA-Seq, the fraction of features used and not the number of neighbours was the primary contributor to memory usage. However, the memory consumption stayed under 10 GB even when ~25% of peaks were included in the analysis. UMAP embeddings labelled with author-determined cell types (Fig. 2b) revealed that the UMAP embedding generated by Scarf accurately captured the heterogeneity of the cell types and categorised cells based on their tissue of origin. Furthermore, UMAP embedding and clustering of cells were not affected by batches of data generation (Fig. 2c).

### Topology-assisted cell downsampling using Scarf

**Fig. 3:**
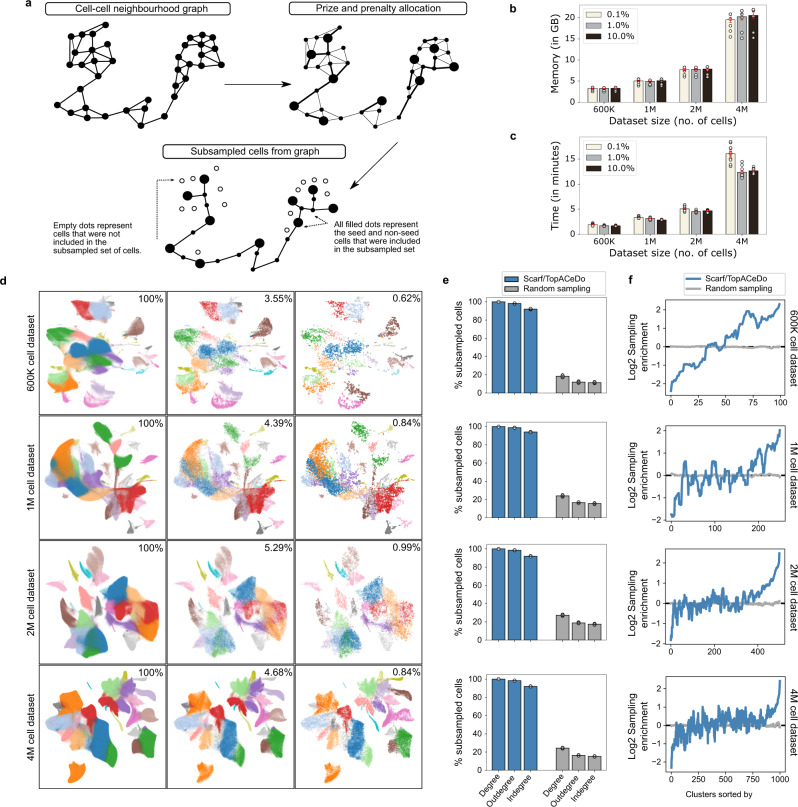
Subsampling cells from large datasets using Scarf. **a** An illustration of the concept behind the cell subsampling, performed using Scarf. Each subplot represents the cell-cell neighbourhood graph computed using Scarf. The nodes in each graph indicate the cells in the dataset. The edges connect two cells if at least one of them is present in the other’s *k* nearest neighbour list. Memory (**b**) and time (**c**) consumption of TopACeDo in four atlas scale datasets. The *x*-axis represents the number of cells in each dataset. Each bar indicates mean value and the error bars indicate standard deviation computed using ten iterations of subsampling (individual datapoints shown as empty circles). The on-figure legends show that downsampling was performed with a maximum sampling rate set at 0.1%, 1% and 10%. **d** Scatter plots showing UMAP embedding of the four atlas-scale datasets. For each of the datasets, UMAPs with all the cells, or cells subsampled with a maximum sampling rate of 10% and 1% are shown. The effective sampling percentage is indicated on each subplot. The cell colours indicate the cluster identities of cells obtained using Leiden clustering. **e** Percentage of subsampled cells that have non-zero graph degrees. Maximum sampling rate for TopACeDo set at 1% and the same number of cells subsampled with TopACeDo was used for random sampling. Bars represent mean and error bars indicate standard deviation obtained using 10 runs of subsampling (individual datapoints shown as empty circles). **f** Plots (a subplot for each of the four datasets) showing the change in cluster enrichment, i.e. change in the proportion of cells from each cluster before and after clustering. The *y*-axis is Log2 cluster enrichment, values below 0 indicate that the proportion of cells from a cluster decreased (depleted) post subsampling and those above 0 indicate that the proportion of cells from clusters have increased (enriched). The values for each cluster represent the mean of 10 iterations. The numbers on the *x*-axis indicate cluster ID and clusters are ordered in decreasing order by size.

Even though Scarf offers memory-efficient processing of single-cell data, including the generation of heterogeneity maps, other downstream analyses often require memory-consuming software that cannot handle a large number of cells. These datasets have prompted a need for downsampling (subsampling, hereon) to allow advanced downstream analysis. Random sampling is often used as a subsampling solution; however, it provides no guarantees of preserving archetypes or differentiation trajectories in the subsampled versions of the data. Recently, GeoSketch^20^ has been proposed to solve this problem, however, GeoSketch is not memory efficient as it has been designed to be run on large-scale computational resources. Here, a subsampling algorithm called TopACeDo (Topology Assisted Cell Downsampling) is embedded into Scarf. TopACeDo leverages the neighbourhood graph structure of the cells to perform downsampling that conserves rare cell types, reduces the proportion of highly represented cell types and preserves the underlying manifold of the transcriptional space. Briefly, TopACeDo identifies landmark points in the graph (seed cells) and then tries to find paths to connect those seed cells using a prize-collecting Steiner tree algorithm (PCST) (Fig. 3a). An implementation of PCST with near-linear time complexity^21^ is used to achieve fast and scalable subsampling on millions of cells (see Methods for details).

Importantly, we found that Scarf was able to perform subsampling on datasets with up to 4 million cells using less than 20 GB of RAM (Fig. 3b). In addition, subsampling took less than 3 min on the 1 million cell dataset and less than 15 min in the case of the 4 million cell dataset (Fig. 3c). UMAP visualisation of four atlas scale datasets indicates that even with subsampling to ~1% of cells, cells belonging to all clusters across the UMAP space were sampled (Fig. 3d).

Furthermore, for quantitative analysis of subsampling, we analysed the degree of connections subsampled cells make with other subsampled cells in the original neighbourhood graph. A high frequency of zero-degree values indicates that many cells are disconnected from other subsampled cells and is a marker of poor subsampling, indicating that intermediary cell states are missing in the subsampled set. When comparing the number of disconnected cells between Scarf with randomly subsampled cells from four atlas-scale datasets, 100% of Scarf-subsampled cells displayed non-zero degree values across all datasets, while random sampling resulted in non-zero degree values in 18.9–26.9% of cells (Fig. 3e).

Two of the primary objectives of subsampling are to decrease redundancy in the dataset and preserve rare cell types/states. These two objectives can be accessed by calculating the change in the proportion of cells from each cluster after subsampling. We show that across the four atlas scale datasets, Scarf was able to reduce the proportion of cells from larger clusters while simultaneously increasing the proportion of cells from smaller clusters (Fig. 3f). The proportion of cells from the smallest cluster in each of the datasets increased between 8.13 and 16.82 folds while the proportion of cells from the largest cluster decreased between 3.35 and 5.26 folds. In comparison, random sampling did not show any increase in the proportion of smaller clusters beyond 1.5 fold or a decrease in the proportion of larger clusters beyond 1.01 fold. As a result, random sampling has a low probability to sample rare clusters. For example, the smallest cluster in the atlas-scale datasets had none of the cells sampled in 20% (1 M cells dataset), 40% (2 M cells dataset) and 20% (4 M cells dataset) (*n* = 10) of random samplings. In contrast, all clusters were sampled using Scarf, regardless of the original cluster size.

**Fig. 4:**
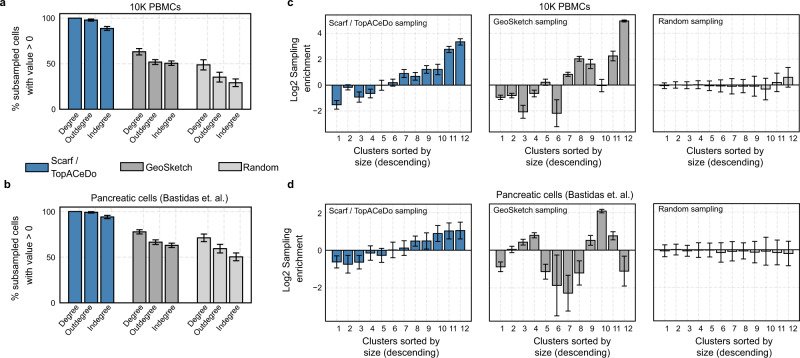
Comparison of Scarf, GeoSketch and random sampling on small-scale datasets. **a** Percentage of subsampled cells from 10 K PBMC dataset and **b** differentiating pancreatic cells that have non-zero graph degrees. Max sampling rate for TopACeDo set at 1% and the same number of cells as subsampled with TopACeDo was used for random sampling and GeoSketch. Bars represent mean and error bars indicate standard deviation obtained using 100 runs of subsampling. **c** Bar plots indicating Log2 cluster enrichment for 10 K PBMC dataset and **d** differentiating pancreatic cells obtained using TopACeDo, GeoSketch and random sampling. Bars represent mean and error bars indicate standard deviation obtained using 100 runs of subsampling. The cluster IDs (ordered by decreasing size) are indicated on the *x*-axis of each subplot.

In order to compare the results of subsampling between Scarf and GeoSketch, we used two contrasting small-scale datasets consisting of either 10 K PBMC cells of distinct cell types (provided by 10X Genomics) or 3.5 K pancreatic cells^22^ within a continuum of differentiation. Visualisation of progressively increasing levels of subsampling on each of these datasets showed that Scarf was able to capture the cells throughout the UMAP landscape (Supplementary Fig. 3). Running 100 iterations of subsampling with either Scarf, GeoSketch or random sampling, 100% of subsampled cells selected by Scarf had a non-zero degree on both the datasets, while the same measurement for GeoSketch was 66.9% and 75.9%, and for random sampling 49.5% and 70.3% in the PBMC and pancreatic cell datasets, respectively (Fig. 4a, b). Compared to random sampling, both Scarf and GeoSketch sampled an increased proportion of cells from smaller clusters and a reduction in the proportion of cells from larger clusters (Fig. 4c, d). For both the datasets, sampling enrichments obtained using Scarf were strongly correlated with decreasing cluster size; pancreatic cells (Pearson’s *r* = 0.97, *p* value=3.36e − 0.7) and PBMC dataset (Pearson’s *r* = 0.94, *p* value=4.96e − 0.6), while in the case of GeoSketch, there was either no correlation (pancreatic cell dataset: Pearson’s *r* = 0.08) or moderate correlation (PBMC data: Pearson’s *r* = 0.77). This indicates that Scarf achieves a consistent reduction in redundancy and increases the representation of rare cells in subsampled datasets.

### Memory efficient projection of cells for compositional analysis and co-embedding of cells

**Fig. 5:**
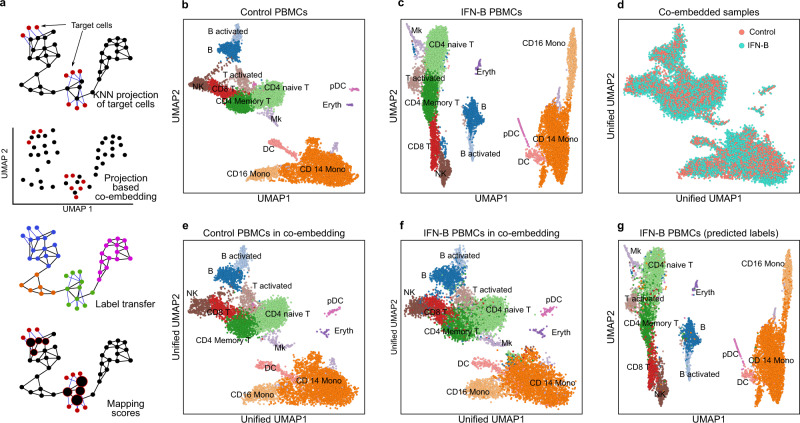
Mapping and co-embedding of cells across datasets. **a** The illustration shows how Scarf uses a KNN projection method to co-embed projected cells into a UMAP space, then, transfer labels to projected cells and assign mapping scores to the reference cells. **b** Scatter plot showing UMAP embedding of untreated and **c** IFN-beta treated PBMCs with cells coloured based on their cluster identity; inferred cell types of each cluster are indicated. **d** UMAP co-embedding of IFN-beta treated PBMCs post projection onto untreated PBMCs. Cells are coloured based on treatment conditions. **e** Plot showing inferred cell types of control PBMCs in the co-embedding obtained upon the projection of IFN-B treated PBMCs. **f** Plot showing inferred cell types of IFN-beta treated PBMCs in the co-embedding obtained upon projection onto control PBMCs. **g** UMAP embedding of IFN-beta PBMCs only; cells are coloured to indicate the cell types obtained through label transfer from control PBMCs upon projection.

The continuous growth of large publicly available single-cell genomic datasets prompts a need for memory-efficient, reliable and appropriate data integration. In Scarf, integration takes a reference-based data alignment approach^15,23,24^ wherein the transcriptional states of query/target cells are interpreted considering the heterogeneity of the reference cells. To achieve reference-based mapping, Scarf implements a highly memory-efficient KNN mapping approach (Fig. 5a; see Methods). This entails that Scarf does not attempt to perform batch correction like other data integration methods^25–27^ but rather brings the target cells into the estimated manifold of the reference cells.

Demonstrating the efficiency and accuracy of Scarf’s data integration approach, we performed mapping of published single-cell RNA-Seq dataset of interferon beta (IFN-β) treated peripheral blood mononuclear cells (*n* = 10,111) to their culture-matched control cells (*n* = 8487)^28^. Before mapping, the datasets were independently analysed to generate UMAP embeddings of both treatment arms (Fig. 5b, c) and cluster annotation was performed using known marker genes of cell types previously reported for this dataset^26^. The annotation was additionally confirmed by performing a marker gene search (Supplementary Fig. 4a,b).

Next, the IFN-β cells were mapped onto the control cells by including the CORAL^29^ correction step (see Methods) and the reference cell-cell neighbourhood graph, now spiked with target cells, was subjected to the UMAP algorithm to obtain a low-dimensional co-embedding including both data sets (see Methods). The ‘unified’ UMAP of the control and IFN-β treated PBMCs (Fig. 5d) shows that the cells from the two datasets are well integrated. We noted that the reference cells in the unified UMAP (Fig. 5e) had a very similar layout as compared to the UMAP resulting from the reference cells alone. This indicates that the inclusion of the IFN-β treated cells did not disrupt the manifold of the control cells. Visualisation of the cell type identity of the mapped IFN-β cells on the unified UMAP clearly showed that Scarf was able to co-embed cell types from the two datasets accurately (Fig. 5f).

Scarf uses the KNN mapping to transfer labels from reference to target cells (see Methods). The original UMAP of IFN-β cells (Fig. 5g) annotated with predicted cell types clearly shows that the label transfer was highly accurate (Supplementary Table 1). Of note, Scarf predicted cell type with high accuracy even on low-abundant cell populations within the dataset. For example, 91.9% and 97.6% of the predicted cell-type labels for pre-dendritic cells and erythrocytic IFN-β treated cells respectively, were true.

**Fig. 6:**
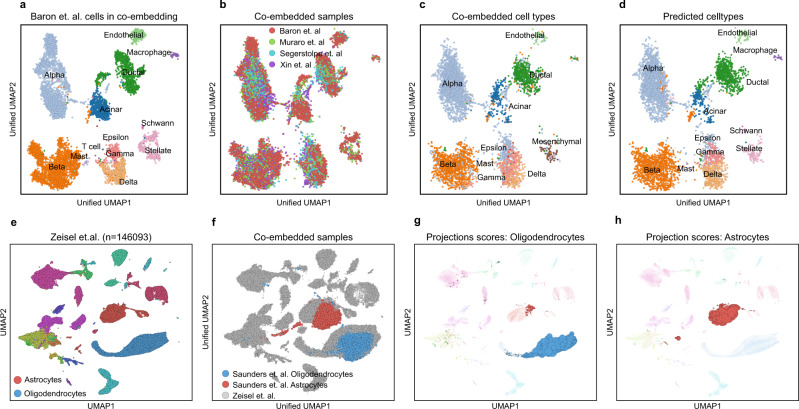
Co-embedding multiple datasets and cell type-specific mapping with Scarf. **a** Scatter plot showing pancreatic cells from Baron et al. in the co-embedding space that was created using the same cells as reference. The projected cells are not shown here, and the reference cells have been coloured according to author annotated cell types. **b** Co-embedding of cells after the projection of cells from three datasets over cells from Baron et al. **c** Cells from the three projected datasets in the unified UMAP space. Cells are coloured based on author-annotated cell types. **d** Cells from the three projected datasets were coloured based on label transfer from the reference cells (Baron et al.) upon projection. **e** UMAP embedding of cells from murine nervous system atlas (Zeisel et al.), cells are coloured based on author annotated cell types. **f** Oligodendrocytes and astrocytes from Saunders et al. co-embedded with cells from Zeisel et al. UMAP plots showing cells from Zeisel et. al. with the size of cells scaled to show mapping score obtained upon the projection of **g** oligodendrocytes **h** and astrocyte from Saunders et al.

Reference-based data integration using Scarf is not limited to two samples. Multiple samples can be co-embedded into the reference manifold simultaneously, here demonstrated using four datasets of pancreatic cells^30–33^. These data are from four different labs, and derived using four different single-cell RNA sequencing platforms viz., InDrop^30^ (*n* = 7715), CEL-Seq2^31^ (*n* = 1946), SMART-Seq2^32^ (*n* = 1809) and C1-IFC SMARTer^33^ (*n* = 1446). Each dataset was processed individually, and the heterogeneity was explored by generating individual UMAP embeddings (Supplementary Fig. 5a–d). Subsequently, we used author-provided cell type labels to annotate the cells. Choosing the InDrop dataset as a reference, we mapped the other datasets using CORAL corrected values and generated a unified UMAP, as done above for the PBMCs. Again, the UMAP embedding of the reference cells remained largely unaltered when co-embedded with the other datasets (Fig. 6a) Moreover, the co-embedding showed mixing of the datasets into each of the reference clusters (Fig. 6b) and visualisation of the cell type identity of the mapped cells on the unified UMAP revealed that co-embedding was highly cell type-specific (Fig. 6c). As previously described for PBMCs, the mapped cells were accurately classified into reference cell type labels (Fig. 6d, Supplementary Table 2–4). For example, a rare population of endothelial cells from CEL-Seq2 (*n* = 19) and SMART-Seq2 (*n* = 15) datasets was labelled to 100% accuracy by the classifier while alpha cells, the most abundant cell type, were identified with >99.8% accuracy in each of the three mapped datasets.

Having assessed the accuracy of the data integration, we aimed to access the scalability of Scarf to larger datasets. Here, we chose a modestly large dataset with 146,093 cells from the mouse nervous system^34^. The dataset was processed to generate a UMAP and annotated with user-provided cell type specification (Fig. 6e). Next, 43,954 oligodendrocytes and 21,885 astrocytes from another brain cell atlas^35^ were mapped to this reference dataset. By utilising the nearest neighbour index that was already generated for the reference, the mapping took less than 30 s and was done under 500MB of memory consumption. Convincingly, the unified UMAP showed that the mapped cell types colocalize with the equivalent clusters in the reference dataset (Fig. 6f). KNN classification was able to accurately predict the correct cell type for 93.7% of astrocytes and 99.5% of oligodendrocytes. Generating co-embedding of large datasets can be time-consuming when multiple mappings on multiple large datasets are performed. To this end, Scarf provides an additional alternative in the form of mapping scores^36^ (see Methods). Here, we demonstrate individually generated mappings for astrocytes and oligodendrocytes, visualised by increasing cell size in proportion to their mapping score (Fig. 6g,h).

### Memory efficient hierarchical clustering and t-stochastic neighbour embedding for single-cell genomics data

Visualisation of single cells in two/three-dimensional space is one of the central tenets of single-cell data analysis. Since single-cell datasets contain tens to hundreds of thousands of features, non-linear dimension reduction algorithms have been considered ideal solutions for 2D/3D data visualisation. Historically, the most popular choice for a variety of single-cell datasets has been t-SNE^37^, while UMAP^9^ is the most common current method for visualisation on account of its computation efficiency (runtimes) and improved preservation of global structure in the data. Alternatively, FIt-SNE^38^ is an improved t-SNE algorithm with runtimes on par with UMAP for larger-scale datasets. Recently, a novel t-SNE algorithm, SG-tSNE^10^, that can directly operate on stochastic KNN graphs was introduced. The implementation of SG-tSNE was shown to have improved runtimes over FIt-SNE and, to be scalable to the three-dimensional embedding of large-scale datasets. However, until our current work, it was unknown, how the embeddings obtained using SG-tSNE compared to those from UMAP and how SG-tSNE performs on a wider selection of datasets.

**Fig. 7:**
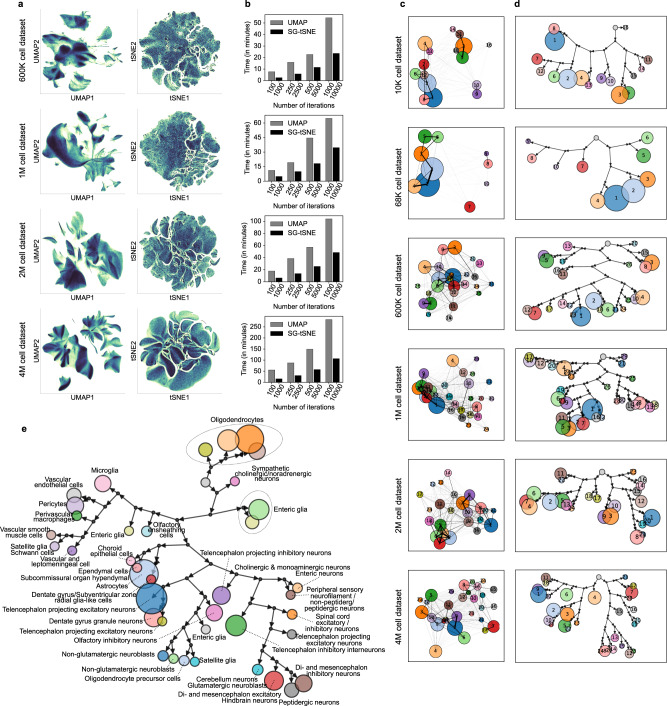
SG-tSNE and Paris hierarchical clustering on single-cell datasets. **a** UMAP and SG-tSNE embeddings of the four, atlas scale single-cell RNA-Seq datasets. The colour gradient shows regions of high (darker regions, with black at the extreme) and low cell density (lighter regions with light yellow at the extreme). **b** Runtime (in minutes) of UMAP and SG-tSNE at a different number of iterations, on Scarf computed cell-cell neighbourhood graph. **c** Scatter plots, for each dataset, show centroids of each Paris cluster in UMAP space. The lines connecting pairs of cluster centroids indicate the weighted sum of edges (in the cell-cell neighbour graph) shared by those clusters. Thicker lines indicate a larger degree of similarity between the clusters. The number on cluster centroids indicate cluster ID. **d** Paris dendrograms of each of the datasets. Each terminal node in the dendrogram represents a cluster of cells (same cluster identities as in C above). The size of each cluster node is set proportionate to the number of cells in that cluster. Each binary branchpoint in the dendrogram is shown with a black circle. The root node is shown as an unlabelled grey node and does not have an incoming arrow. **e** Paris dendrogram of the mouse nervous system cell atlas (Zeisel et al.). The coloured nodes indicate a Paris cluster and are labelled with cluster IDs. The nodes are sized proportionately to the number of cells in the cluster. The author annotated cell types present in each cluster are indicated next to the cluster node.

The neighbourhood graph computed by Scarf can be directly fed into either UMAP or SG-tSNE algorithm. Furthermore, Scarf uses the same initial embedding for both UMAP and SG-tSNE (see Methods), thus restricting the differences between the two methods to the underlying algorithm itself and the parameters used. We benchmarked the run-time of UMAP and SG-tSNE on four atlas scale datasets of 600 K, 1 M, 2 M and 4 M cells at multiple iterations (see Methods for details). Both algorithms run iteratively for a user-defined number of steps. 2D UMAP (250 iterations) and SG-tSNE (2500 iterations) plots were obtained from the four atlas scale datasets (Fig. 7a). SG-tSNE consumed less time for 2500 iterations compared to UMAP’s 250 (5.8, 9.9, 13.6 and 30.7 min vs 15.9, 19.3, 38.5 and 87.6 min, for the 600 K, 1 M, 2 M and 4 M cell datasets respectively). Moreover, we observed that for the corresponding iterations across datasets, SG-tSNE scaled better to the increasing number of cells; for 4 M cell datasets the runtime of 10,000 iterations SG-tSNE (96.45 min) was similar to the runtime of UMAP with 250 iterations (Fig. 7b).

Annotation of UMAP (Supplementary Fig. 6a) and SG-tSNE (Supplementary Fig. 6b) plots with Leiden clusters resulted in well-separated clustering in both cases and the relative distances between clusters could be easily visualised on the UMAP plot. Moreover, the clusters were positioned in a similar vicinity to other clusters in both embeddings. To quantify this, we calculated the Spearman rank correlation between the nearest neighbour across the clusters in the embedded space and the original cell–cell neighbourhood graph (Supplementary Fig. 6c). The spearman correlations coefficient values across the clusters show that UMAP was able to learn cluster relations quickly and showed only a slight improvement from the increased number of iterations. In all four datasets, SG-tSNE showed a lower mean Spearman’s rho value than UMAP at a fewer number of iterations but improved to be higher than UMAP when a larger number of iterations were used. For example, in the case of the 4 M cell dataset, the SG-tSNE’s value increased from 0.75 (1000 iterations) to 0.93 (10,000 iterations).

Next, we compared UMAP and SG-tSNE for the preservation of local data structure by quantifying the number of KNN neighbours found in the vicinity of each cell in the UMAP or SG-tSNE space (Supplementary Fig. 6d). Across the four datasets, SG-tSNE led to improved local structure with an increasing number of iterations while no benefit was observed for UMAP embeddings. UMAP, with 1000 iterations, had 0.15, 0.23, 0.24 and 0.3 mean fractions of nearest neighbours preserved in the embedding of 600 K, 1 M, 2 M and 4 M cell datasets, respectively. SG-tSNE, on the other hand, at 10,000 iterations, reached the values of 0.41, 0.51, 0.5 and 0.46, in the respective datasets.

Clustering is one of the most critical aspects of single-cell data analysis wherein cells are partitioned into distinct groups. Hierarchical clustering^39–42^-based methods can provide accurate partitioning of cells and discover rare cell types in the data but are not scalable to atlas size datasets^43^. Alternatively, Graph clustering^44–47^ methods have become a popular choice for clustering on single-cell data and Louvain^46^ and Leiden^44^ algorithms are routinely used through Seurat^15^ and Scanpy^11^ packages. However, unlike hierarchical clustering methods, Louvain and Leiden are unable to reflect the relationship between clusters. Scarf performs clustering using the highly scalable Leiden algorithm. Like other graph-based methods and unlike hierarchical clustering, Leiden is unable to explicitly indicate the relationships between clusters. PAGA^48^ was introduced as a method that can explicitly indicate a relationship between clusters once created by any clustering method. Unfortunately, it can become hard to interpret those relationships on large datasets where multiple clusters with complex connection patterns are present (Fig. 7c). Within Scarf, we introduce a recently published approach, Paris^8^, that has not previously been applied to single-cell genomics data. Paris is a graph clustering approach that rather than creating flat clusters of cells, creates a dendrogram of cells akin to hierarchical clustering methods.

Specifically, Paris computes a binary tree representing the various nested clusters of the graph, at different levels of granularity. The top-level clusters give the core structure of the graph while the lower-level clusters provide the local structure. Like Louvain and Leiden, Paris is a scalable algorithm applicable to large graphs with millions of cells. High performance is achieved through the nearest neighbour chain algorithm, where pairs of clusters to be merged are locally searched, following the edges of the graph. The successive merged clusters are stored and aggregated to compute the final binary tree.

We applied Paris to the six datasets used previously (Supplementary Fig. 7a). In order to allow comparison with Leiden clustering (Supplementary Fig. 7b), we cut the Paris dendrogram to obtain the same number of clusters as Leiden clustering. Coalesced Paris dendrograms presented a clear structure of cluster relationships (Fig. 7d) and the mean concordance between the Paris and Leiden clusters was found to be between 84.9% and 93.1% across the datasets (Supplementary Fig. 7c). These values were significantly larger (*p* value < 9.18e − 11; Mann Whitney U test) than randomly shuffled cluster identity wherein mean concordance with Leiden ranged between 9.06% and 29.3%.

To ascertain the biological meaningfulness of Paris dendrograms, we used scRNA-Seq data from cells of the murine nervous system^34^. The authors manually curated multiple taxonomies of cell types in the cell atlas. Importantly, Paris was able to retrace the taxonomic relationships between the cell types (Fig. 7e) presenting a dendrogram with relevant and clearly separated branches for vascular, immune, glial and neuronal cell types.

Together, these results show that Scarf can perform all critical analysis steps of single-cell genomics data including filtering, normalisation, feature selection, linear dimension reduction, neighbourhood graph creation, embedding using UMAP and t-SNE, clustering, downsampling and cell projection on even the largest available data sets on a regular laptop in a time-efficient manner. Importantly, the resulting data are consistent or improved compared to using the memory-exhausting current state-of-the-art tools.

## Discussion

The means to handle and process large-scale single-cell genomics data using readily available hardware are urgently needed. Most atlas-scale projects resort to providing online interfaces with UMAP/t-SNE plots over which users visualise the expression of different genes. But this can be grossly insufficient for many research tasks. Researchers often generate datasets that need co-analysis with other atlas-scale datasets which necessitates the use of high-performance computing machines. Another common-usage scenario where researchers need to process count matrices of large-scale datasets is when performing custom sub-selection of cells and generating new UMAP/t-SNE and clustering. Feature selection on a sub-selection of cells can improve the resolution of UMAP/t-SNE and increase the sensitivity of rare cell-type and cell-state identification. We designed Scarf to give researchers the ability to analyse and re-analyse atlas-scale datasets on their laptop computers.

Coupled with memory-efficient methods such as Kallisto Bustools^49^ which generates cell-gene (or cell-transcript) count matrices, Scarf represents an end-end solution for the analysis of single-cell RNA-Seq datasets. During the preparation of this manuscript an R-based memory-efficient tool, ArchR^50^, was published for analysis of scATAC-Seq data. In the paper describing this feature-rich tool, the authors benchmarked a simulated PBMC dataset with 1.2 million cells and found the memory usage to be over 20 GB in a small infrastructure setting. With Scarf, we were able to perform corresponding steps in a scATAC-Seq dataset with 700 K cells within 5 GB RAM. ArchR needs genome-aligned fragment files and unlike Scarf, does not support direct input of precalculated cell-peak matrices that are readily available for many atlas scale datasets.

Our subsampling algorithm is designed to leverage the fact that a cell-cell neighbourhood graph is already calculated in a memory-efficient way. The obtained subsampled set can thereby be exported and used with external tools to apply methods that are not currently directly supported in Scarf. We allow seamless import and export of data between Scarf and Scanpy. Though our comparison metrics indicate that Scarf performs better than GeoSketch, we want to highlight that GeoSketch does not require clustering information that Scarf/TopACeDo needs to perform subsampling.

As previously described^51^, the embedding obtained from t-SNE and UMAP can be sensitive to the parameters used in the analysis. Hence, one method is not necessarily better than the other, but rather complementary techniques for visualisation. Without extensive parameter tuning, SG-tSNE can generally reveal the heterogeneity of large-scale datasets more clearly than UMAP, while on the other hand UMAP can be more useful to investigate the relationship between clusters and visualise biological processes like differentiation. Our contribution here was to perform a head-to-head comparison of UMAP and a graph-based t-SNE (SG-tSNE) on multiple datasets by providing the same KNN graph and coordinate initialisation.

In Scarf, we introduce a graph-based hierarchical clustering method that can highlight cluster similarity. Single-cell specific hierarchical clustering algorithms have been shown to not be scalable to large-scale datasets^42^. Our benchmarks showed that the Paris algorithm applied to the Scarf computed cell-cell neighbourhood graph creates a full dendrogram of the 4 M cell dataset in 130 min (Supplementary Fig. 7d, e). This runtime is much slower compared to Leiden clustering which generated a fixed resolution in less than 5 min. However, Paris reveals a full profile of cell-cell relationships while Leiden provides only static partitioning using an arbitrary ‘resolution’ parameter. In practice, analysts often run the Leiden algorithm with many different values for the ‘resolution’ parameter to ascertain an optimal number of clusters. In contrast, the creation of a cell dendrogram in Paris is parameter-free and can subsequently be cut into any desired number of clusters within a few seconds. Hence, the effective runtime of the Leiden algorithm can be longer than Paris, especially on large and complex datasets.

Taken together, Scarf represents a comprehensive toolkit for the analysis and integration of even the largest single-cell datasets on desktop computers, making single-cell genomics available to a significantly larger segment of the research community while simultaneously and significantly driving down the computational cost.

## Methods

### Design principles

#### Data storage that supports parallel processing

Scarf, though primarily intended for memory-efficient analysis, provides enhanced parallel processing capabilities for single-cell genomics analysis. We chose the Zarr file format^12^ for on-disk representation and storage of data. The benefit of using Zarr over HDF5 (used by Loom and Anndata) is its ability to perform concurrent read/write operations on the data. We use the Dask^52^ library to load and perform efficient concurrent operations on the Zarr-backed data matrix. Using the Blosc library we were able to efficiently compress the data matrix while still in dense format. Storing and loading compressed, chunked dense matrix allowed us to avoid sparse/dense interconversions, a strategy used by other packages to manage memory^11,26^. The size of the chunks of the count matrix saved by Zarr represents the trade-off between speed and memory efficiency. Larger chunks allow faster processing by reducing loading cycles but have a larger memory footprint than the smaller chunks. Individual chunks are processed and fed into downstream algorithms in parallel.

#### Efficient usage of CPU resources

We observed that in the Python ecosystem many libraries aggressively use all the available CPU cores. Though this is usually not an issue when running analysis on servers, it can seriously impediment users’ ability to simultaneously use their laptops/desktops if all CPU cores are being used. Hence, we have extensively placed CPU usage restrictions throughout the code so that only a user-determined number of CPU cores are engaged during the analysis. We have further enabled support for running non-linear dimension reduction methods, which can often be the longest-running step in the pipeline, on multiple CPU cores.

#### Optimisations for interactive iterative analysis

Single-cell genomics analysis has several parameterised steps and estimating the optimal parameters can often be difficult beforehand. Hence, users explore their datasets with different combinations of parameters. Scarf aggressively caches all the intermediate data generated during analysis workflow. This avoids unnecessary re-computation.

#### Multi-assay support

Multi-omics experiments like CITE-Seq are well supported by Scarf. Scarf can store assays data from each of the modalities under a separate branch of the Zarr hierarchy. The cell level attributes are stored outside the assays while feature level attributes and assay’s count matrix is stored under the assay group. Scarf’s programmatic interface mimics this hierarchy. Also, Scarf assigns one of the assays as default when the dataset is loaded, and all the functions will act on this default assay. Almost all the functions associated with the *DataStore* object use the *from_assay* parameter which can be used to assign the assay for the operation.

### Cell filtering

Poor quality cells can be filtered based on any cell attribute. Scarf has a *auto_filter_cells* function that will model a normal distribution of a chosen cell attribute and then remove cells with value probability below x or over 1 − *x*, wherein *x* is a user-selected cut-off value (default value: 0.01). Users can also apply custom filtering methods to remove or include cells in the analysis.

### Count normalisation

For scRNA-Seq datasets, Scarf performs library size normalisation of the cells. Hence, the normalised value of a gene/feature in a cell ${y}_{{Fc}}$ can be calculated as

$${y}_{{fc}}=S.\frac{{x}_{{fc}}}{\mathop{\sum}\nolimits_{{f}^{{\prime} }{{{{{\rm{\epsilon }}}}}}F}{x}_{{f}^{{\prime} }c}}$$

where *F* is the feature set, *c* represents a cell and *S* is a scaling factor. The normalised value can optionally be transformed into log scale: ${y}_{{fc}}={{\log }}\left(1+{y}_{{fc}}\right)$

For scATAC-Seq datasets, TF-IDF (term frequency-inverse document frequency) normalisation is performed.

$${y}_{{fc}}=\frac{{x}_{{fc}}}{{n}_{{F}_{c}}}.{{\log }}\left(1+\frac{{N}_{C}}{{n}_{{C}_{f}}}\right)$$

where ${n}_{{F}_{c}}$ represents the total number of accessible peaks (those with non-zero values) in a given cell, ${n}_{{C}_{f}}$ represents a total number of cells where a given peak is accessible. ${N}_{c}$ is the total number of cells in the dataset.

### Feature selection

For scRNA-Seq datasets, Scarf provides the highly variable gene (HVG) selection approach as previously reported^53^. For this purpose, the mean expression and variance of genes are calculated across all the cells and are log-transformed. The next step is to remove the mean-variance trend in the feature space. For this, genes are divided into equal-sized bins based on their mean expression value; from each bin, the gene with the lowest expression value is selected. A lowess curve (using Python’s statsmodels package^54^) is fitted to the selected genes. The fitted curve is used to predict the ‘expected variance’ for each gene using mean expression as the independent variable. The ‘expected variance’ is subtracted from observed variance to obtain corrected variance based on which HVGs are selected. Optionally, the users can put constraints on mean expression and corrected variance to perform HVG selection. For scATAC-Seq datasets, each peak is assigned a prevalence score. The prevalence score is the sum of TF-IDF normalised values for all the features. Top *n* peaks, sorted by prevalence scores are chosen by users to perform the downstream analysis.

### Primary dimension reduction

For single-cell RNA-Seq datasets, principal component analysis (PCA) is applied. PCA is normally applied to a subset of features prioritised using a feature selection method like highly variable gene selection but can also be applied to all the features or user-designated custom subset of features. In Scarf, Sklearn’s incremental PCA implementation^55^ is used which allows PCA to be trained iteratively using only a subset of cells at a time. Additionally, using Scarf, users can easily use only a subset of cells to train PCA and the rest of the cells can still be transformed into this trained PCA space. The data is standard scaled before performing the PCA. For scATAC-Seq datasets, we use Gensim’s iterative latent semantic indexing (LSI)^56^ method to perform dimension reduction. Similar to PCA, the LSI method can be trained iteratively and hence is scalable to large (aka atlas scale) datasets.

### Cell-cell neighbourhood graph

An approximate nearest neighbour algorithm, the hierarchical small navigable world (HNSW), as implemented in the HNSWlib library is utilised^57^. The dimension-reduced values are used to build the HNSW index. By default, Scarf uses the Euclidean metric as the measure of distances between the cells. The index once trained is saved onto the disk for later use. The nearest neighbour search is then performed using the index, querying for ‘k’ neighbours. During the query, it is noted if cells are indeed the nearest neighbours of themselves and this is summarised and reported as the recall value. The resulting KNN index and the distances to the KNNs are saved onto the disk. Thereafter, an edge normalisation step is performed on the KNN graph wherein the distances are converted into a continuous scale bounded between 0 and 1 using UMAP’s edge weight smoothening algorithm^58^. The resulting adjacency matrix is saved in sparse format on disk. This adjacency matrix represents the graph that is directly fed downstream steps like UMAP, SG-tSNE, Paris and Leiden clustering.

### Calculation of initial embedding

Before running UMAP and tSNE, cells are usually ascribed an initial embedding which can be either random coordinates (default for many t-SNE algorithms) or informed measures such as first to/three principal component^51^ or Laplacian eigenmaps (default for UMAP). In Scarf, the initial embedding for UMAP and tSNE is calculated by fitting the MiniBatch Kmeans algorithm from scikit-learn package^55^ during the same step as the graph construction. This gives a cluster centroid matrix of form *c* *×* *d*; where *c* is the number of Kmeans clusters and *d* is the number of dimensions of PCA used in the graph construction step. Thereafter, another round of PCA is performed on this matrix to two obtain either *c* *×* 2 (for 2D embedding) or *c* *×* 3 (in the case of 3D embedding) matrix. Thereafter, each cell is assigned a 2D or 3D initial embedding coordinate based on its Kmeans cluster identity.

### Benchmarking setup

All benchmarks were performed on a computing cluster comprised of nodes with configuration: 16 2.6 GHz CPUs and 190 GB RAM. Each compute node had a local SSD drive and files were moved to local storage to allow exclusive IO operations for each job. A job comprised of either the Scarf or Scanpy pipeline which was monitored for memory consumption using Linux’s ‘ps’ utility. We used Scarf’s version 0.7.7 for all the analysis. Scanpy’s version 1.6.1 was used for all the benchmarking. Geosketch version 1.2 was used for comparison with TopACeDo.

### Marker feature identification

In Scarf, we adopt a simple and fast approach to identify marker genes based on gene ranks. All the features are ranked based on normalised values. Thereafter, for each gene, we calculate the mean rank for each group/cluster. The mean ranks for each gene cluster are normalised by dividing by the sum of all mean ranks from all the clusters. The sorted list of normalised mean ranks gives the ordered list of marker features for each cluster. The marker scores generated by Scarf are in the range between 0 and 1 with lower scores indicating poor specificity of a marker gene.

### TopACeDO algorithm

The first step in the TopACeDO algorithm is to identify ‘seed’ cells in the graph. For this, two metrics for each cell (referred to as a node in the neighbourhood graph) are calculated: n-neighbourhood degree (NND) and neighbourhood-connectedness (NC). The degree of the node is calculated as the total number of other nodes this particular node is connected to. 1-neighbourhood degree is the sum of the degree of all nodes that are connected to a given node. Hence, NND is computed by iterating neighbours of neighbours over n-step distance and captures the density of the connections around a given node in the graph. The second metric is neighbourhood connectedness which captures if a given number of connections are shared between many or few nodes. To calculate the NC of each cell, the sum of shared nearest neighbour distances (Jaccard distance) between a node and all its neighbours is calculated. Thus, if a node is connected to other nodes that are strongly connected among each other, this node will get a high value for neighbourhood connectedness.

For the next step, the algorithm uses the partitioning of cells. Here, median NND and NC are calculated for each cluster of cells and the median value is used to adjust the sampling rate for each cluster. A higher median NND leads to a reduction in sampling rate while a higher NC leads to a reduced sampling rate and vice versa. Based on the sampling rate, the number of cells to be sampled from each of the clusters are determined. Each cluster is then sub-clustered, wherein the number of sub-clusters is the same as the number of cells to be sampled; one cell is then sampled from each of the sub-clusters. These sampled cells are referred to as ‘seed’ cells.

All the seed cells are assigned a constant prize value. Here, we used a value of 10. The edge penalty ${E}_{p}$ for each edge is calculated as follows:

$${E}_{p}={E}_{{cm}}.{E}_{{bw}}^{-{E}_{w}}$$

wherein, ${E}_{{cm}}$ and ${E}_{{bw}}$ are user-provided parameters, edge cost multiplier and edge bandwidth, respectively and ${E}_{w}$ is the edge weight in the graph. Higher values for ${E}_{{cm}}$ will make reaching remote cells in the graph more difficult but at the same time will discourage the inclusion of non-seed cells in the subsampled set. Higher ${E}_{{bw}}$ accentuates the difference among edge penalties. Here we used ${E}_{{cm}}=1$ and ${E}_{{bw}}=10$.

Once, the prizes on the seed cells and penalties on all the edges are set, we run an approximate implementation PCST algorithm on the cell-cell neighbourhood graph. This implementation can be found here: https://github.com/fraenkel-lab/pcst_fast. We run an unrooted version of PCST and set the *num_clusters* parameter to 1 in order to get one connected Steiner tree for each component of the cell-cell neighbourhood graph.

TopACeDo was run on atlas scales datasets whose graphs were calculated using 21 nearest neighbours(k) and 25 PCA dimensions (calculated on 2500 HVGs).

### KNN mapping and Integrated embedding

#### Normalisation to remove batch effects

The reference-based KNN mapping approach in Scarf overcomes batch effects due to the following reasons. Only highly variable genes from the reference dataset are used for mapping. This means that we use those genes that already capture the heterogeneity of the reference dataset. Hence, the genes that might be contributing to the batch effects are likely to be ignored. The assumption here, as in most other methods like MNNcorrect, is that the batch effect is orthogonal to cellular heterogeneity. Secondly, Scarf performs a second round of normalisation that scales the total expression values from each cell (from reference as well as target datasets) to the same value. This means that the sum of values for all the selected genes (HVGs defined in the reference) from a given cell will always add up to 1000 (default value). The motivation for this is, that if let’s say, technology/batch 1 captures a lot more ribosomal genes than batch 2; then resultantly, the total normalised value for all non-ribosomal will be much lower in batch 1 compared to batch 2. By rescaling, we will be able to remove this difference (assuming that ribosomal genes do not make it to the HVG list, otherwise heterogeneity is not orthogonal to batch effect). Thirdly, specifically related to the last point, in Scarf, we automatically disqualify (this behaviour can be easily overridden by users) mitochondrial, ribosomal and some cell cycle genes from the HVG list as these genes are usually the ones contributing (completely or partially) to batch effects (full list of patterns that are ignored for HVG selection can be found here).

KNN mapping is performed using the precalculated HNSW index of the reference dataset. By avoiding recalculation of the index, Scarf can quickly perform mapping even when cold started on a dataset. If the two datasets have identical populations, we suggest the usage of the domain shift correction method, CORAL, that’s built into Scarf. CORAL^29^ correction is performed as follows:

$${A}_{{Coral}}={Cov}{\left(S\right)}^{1/2}\cdot {Cov}{\left(T\right)}^{1/2}$$

$${T}_{{DC}}={A}_{{Coral}}\cdot T$$

wherein, $S$ and $T$ are scaled and normalised count matrixes of source and target samples respectively and ${T}_{{DC}}$ is the domain corrected matrix of the target dataset. Because the covariance, ${Cov}$, of matrices is calculated across features, this normalisation is easily scalable to large datasets.

#### Calculation of unified UMAP

All reference graphs were constructed with 11 neighbours and using 25 PCA dimensions (calculated on 2500 HVGs). The unified UMAP/SG-tSNE for the reference and target cells are calculated by spiking the reference cell-cell neighbourhood graph with target cells based on their nearest neighbours (top 3 for all the results here) in the reference set. The target-reference edges are assigned a constant weight which users can tune manually. We used a value of 1 for all the results presented. The UMAP was run for 100 iterations on the unified graph of reference and target cells to obtain a unified UMAP embedding.

#### Mapping scores

To calculate the mapping scores of reference cells, we first calculate the edge weights, *W*, between reference and target cells as follows:

$${W}_{{rt}}=1/\left({np}.{\log }\left({D}_{{rt}}+1\right)+1\right)$$

wherein ${D}_{{rt}}$ is the Euclidean distance between a reference and a target cell. For each reference cell, mapping score, ${M}_{r}$ is calculated is as follows:

$${M}_{r}=\frac{\mathop{\sum}\limits_{t\epsilon {K}_{t}}{W}_{{rt}}}{{N}_{T}}$$

wherein ${K}_{t}$ are all the targets that mapped to the given reference cell and ${N}_{T}$ is the number of target cells. Normalising with ${N}_{T}$ allows improved comparison of mapping scores from two different mappings. For the reported results, the mapping scores were additionally multiplied by a scalar value of 1000 and log-transformed.

#### Label transfer from reference to target sample

Label transfer is performed in Scarf using the KNN projection. To ascribe a reference label to a target (projected) cell, we compute the weighted sum of all edges from a target to all the connected reference classes. If the target cell has at least 50% (default threshold and used throughout here) of all the edge weights a single reference class then the target cell is ascribed to that reference class, otherwise, it is labelled as NA (null value meaning not assigned).

### Comparison of UMAP and SG-tSNE on atlas scale datasets

We chose four iteration sets for UMAP (100, 250, 500 and 1000) and ten times for SG-tSNE. We observed that individual iterations of SG-tSNE had lower runtime than UMAP, hence we chose iterations so that they can have comparable runtimes. Both the methods were run in parallel mode using 16 computing cores. The same KNN graphs (generated with *k* = 11, PCA dimensions = 25 and HVGs = 2500) were used as input for both UMAP and SG-tSNE. Parameters used for UMAP: *min_dist* *=* *0.5, spread* *=* *2.0*. Parameters used for SG-tSNE*: alpha* *=* *50, box_h* *=* *1*. To find the KNN of cells in the UMAP/SG-tSNE space, we used the *NearestNeighbors* function from the scikit-learn package with Euclidean distance metric and *KD tree* algorithm. To calculate Spearman’s correlation between the clusters in the neighbourhood graph and UMAP/tSNE space, we took the following approach. We first created a similarity matrix of dimension (*P*, *P*) where *P* is the number of clusters identified in the dataset. This similarity matrix is calculated by calculating the weighted sum of edges (in cell-cell neighbourhood graph) between cells of each pair of clusters. Another similarity matrix is calculated based on the KNN graph calculated on the UMAP/tSNE embedding. Spearman’s correlation coefficient is calculated between each column (cluster) from the two similarities after log2 transforming the values.

### Reporting summary

Further information on research design is available in the Nature Research Reporting Summary linked to this article.

## Supplementary information

Supplementary Information

Reporting Summary

## Data Availability

Following is the list of all the publicly available datasets used in the study. The dataset id, as referenced in this study, are shown in square brackets. The links for the dataset location are provided where datasets were not downloaded from GEO/ArrayExpress. Kang et al.^28^ [kang_15K_pbmc_rnaseq]: GSM2560248 (GEO); Kang et al.^28^ [kang_14K_ifnb-pbmc_rnaseq]: GSM2560249 (GEO); Baron et al.^30^ [baron_8K_pancreas_rnaseq]: GSE84133 (GEO); Muraro et al.^31^ [muraro_2K_pancreas_rnaseq]: GSE85241 (GEO); Segerstolpe et al.^32^ [segerstolpe_2K_pancreas_rnaseq]: E-MTAB-5061 (ArrayExpress); Xin et al.^33^ [xin_1K_pancreas_rnaseq] GSE81608 (GEO); Zeisel et al.^34^ [zeisel_161K_nervous_rnaseq]: https://storage.googleapis.com/linnarsson-lab-loom/l5_all.loom; Saunders et al.^35^ [saunders_110K_brain_rnaseq]: http://dropviz.org/; 10x genomics datasets [tenx_8K_pbmc_citeseq]: http://cf.10xgenomics.com/samples/cell-exp/3.0.0/pbmc_10k_protein_v3/pbmc_10k_protein_v3_filtered_feature_bc_matrix.h5; Bastidas-Ponce et al.^22^ [bastidas-ponce_4K_pancreas-d15_rnaseq]: https://github.com/theislab/scvelo_notebooks/raw/master/data/Pancreas/endocrinogenesis_day15.h5ad; Zheng et al.^16^ [zheng_69K_pbmc_rnaseq]: http://cf.10xgenomics.com/samples/cell-exp/1.1.0/fresh_68k_pbmc_donor_a/fresh_68k_pbmc_donor_a_filtered_gene_bc_matrices.tar.gz; Human Cell Atlas Data Portal [hca_783K_blood_rnaseq]: https://data.humancellatlas.org/project-assets/project-matrices/cc95ff89-2e68-4a08-a234-480eca21ce79.homo_sapiens.mtx.zip; 10x genomics Data datasets [tenx_1.3M_brain_rnaseq]: http://cf.10xgenomics.com/samples/cell-exp/1.3.0/1M_neurons/1M_neurons_filtered_gene_bc_matrices_h5.h5; Cao et. al^17^ [cao_2.1M_moca_rnaseq]: GSE119945 (GEO) https://shendure-web.gs.washington.edu/content/members/cao1025/public/mouse_embryo_atlas/gene_count.txt; Cao et al.^18^ [cao_4.9M_fetal_rnaseq]: GSE156793 (GEO) https://descartes.brotmanbaty.org/bbi/human-gene-expression-during-development/; Domcke et al.^19^ [domcke_721K_fetal_atacseq]: GSE149683 (GEO) https://descartes.brotmanbaty.org/bbi/human-chromatin-during-development/. All count matrices used in this study can be obtained using the following command: s*carf.fetch_dataset(dataset_id)* Ids for all available datasets can be obtained using this command: *scarf.show_available_datasets()*.

## Code availability

The source code for the Scarf package is available here: github.com/parashardhapola/scarf The documentation for installation and usage of Scarf can be found here: scarf.readthedocs.io. The notebooks and scripts used in the paper can be found here: https://osf.io/cbu6a.
