# Supplementary material for: Scarf enables a highly memory-efficient analysis of large-scale single-cell genomics data
Source: Nat Commun. 2022 Aug 8;13:4616. doi: 10.1038/s41467-022-32097-3 (PMC9360040; doi:10.1038/s41467-022-32097-3)
Supplement: Supplementary file 1 — Supplementary Information [file 41467_2022_32097_MOESM1_ESM.pdf]

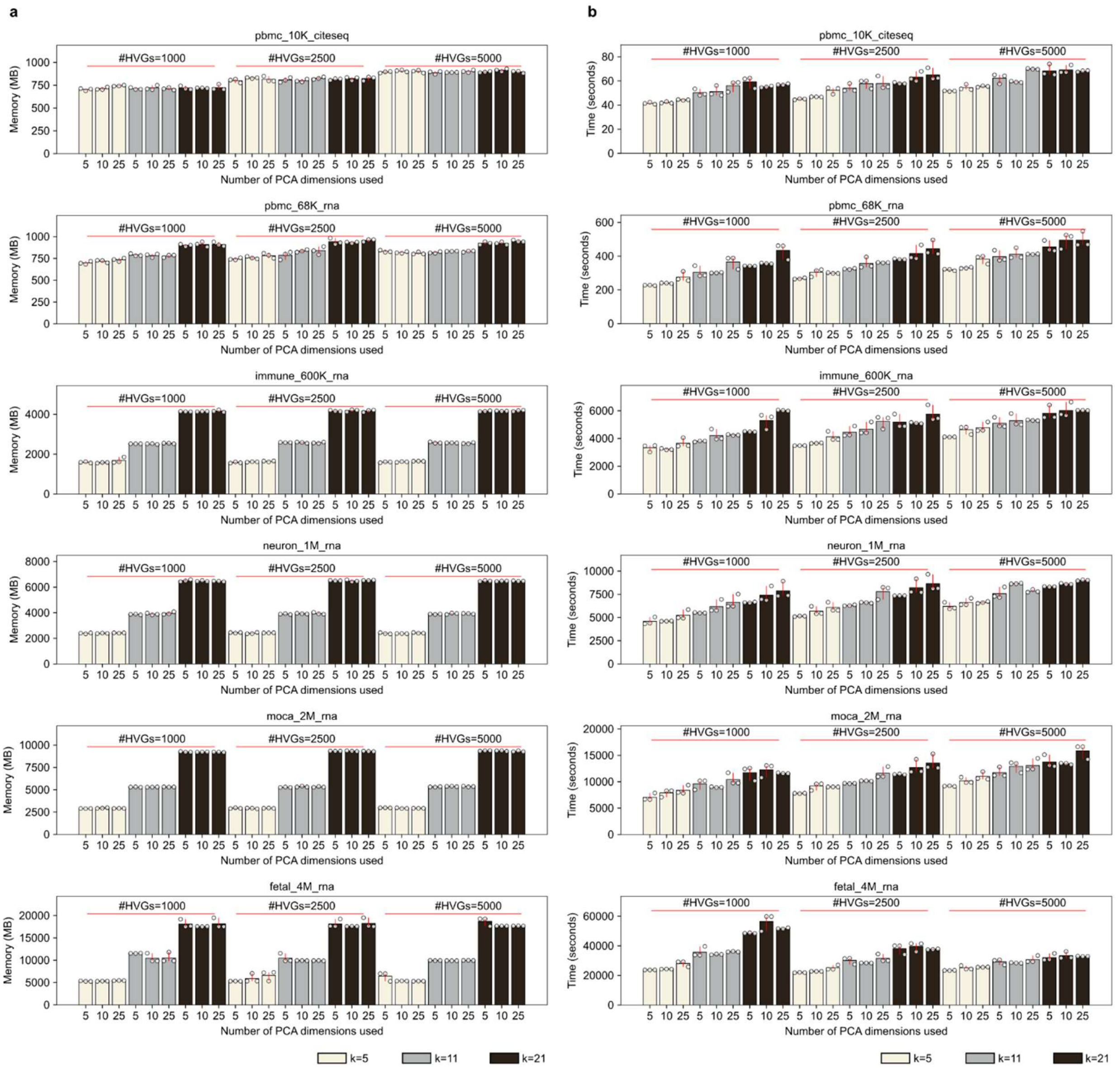

<u>Supplementary Figure 1</u>: Benchmarking memory and time usage of Scarf across different parameters. Bar plots showing memory **(a)** and time **(b)** consumption of Scarf across different datasets used in the analysis. Error bars show standard deviation calculated using three replicates (individual datapoints shown as empty circles). Each bar indicates the time or memory usage under a combination of the number of HVGs used, the value of parameter k (nearest neighbours in KNN graph) and the number of PCA dimensions used to create the KNN graph. Each bar is the mean value, and the error bars indicate standard deviation computed using three technical runs.

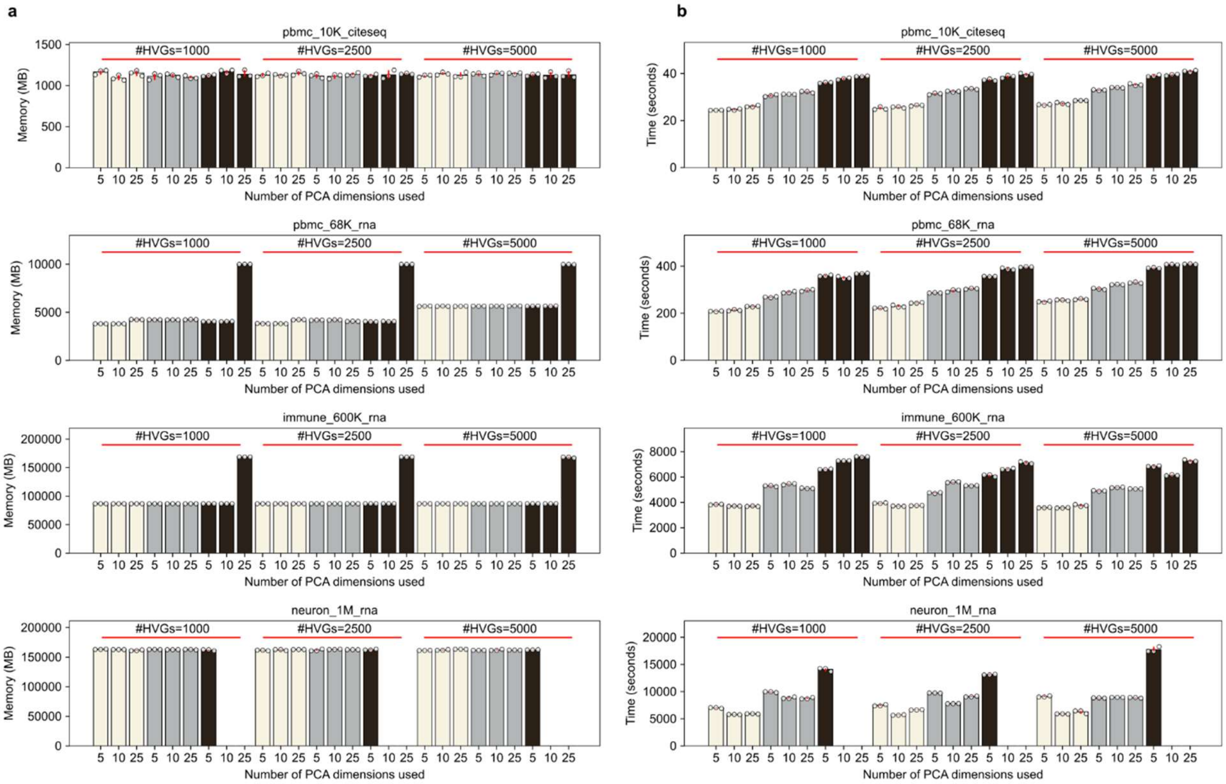

<u>Supplementary Figure 2</u>: Benchmarking memory and time usage of Scanpy across different parameters. Bar plots showing memory **(a)** and time **(b)** consumption of Scanpy across different datasets used in the analysis. Error bars show standard deviation calculated using three replicates (individual datapoints shown as empty circles). Each bar indicates the time or memory usage under a combination of the number of HVGs used, the value of parameter k (nearest neighbours in KNN graph) and the number of PCA dimensions used to create the KNN graph. Each bar is the mean value, and the error bars indicate standard deviation computed using three technical runs.

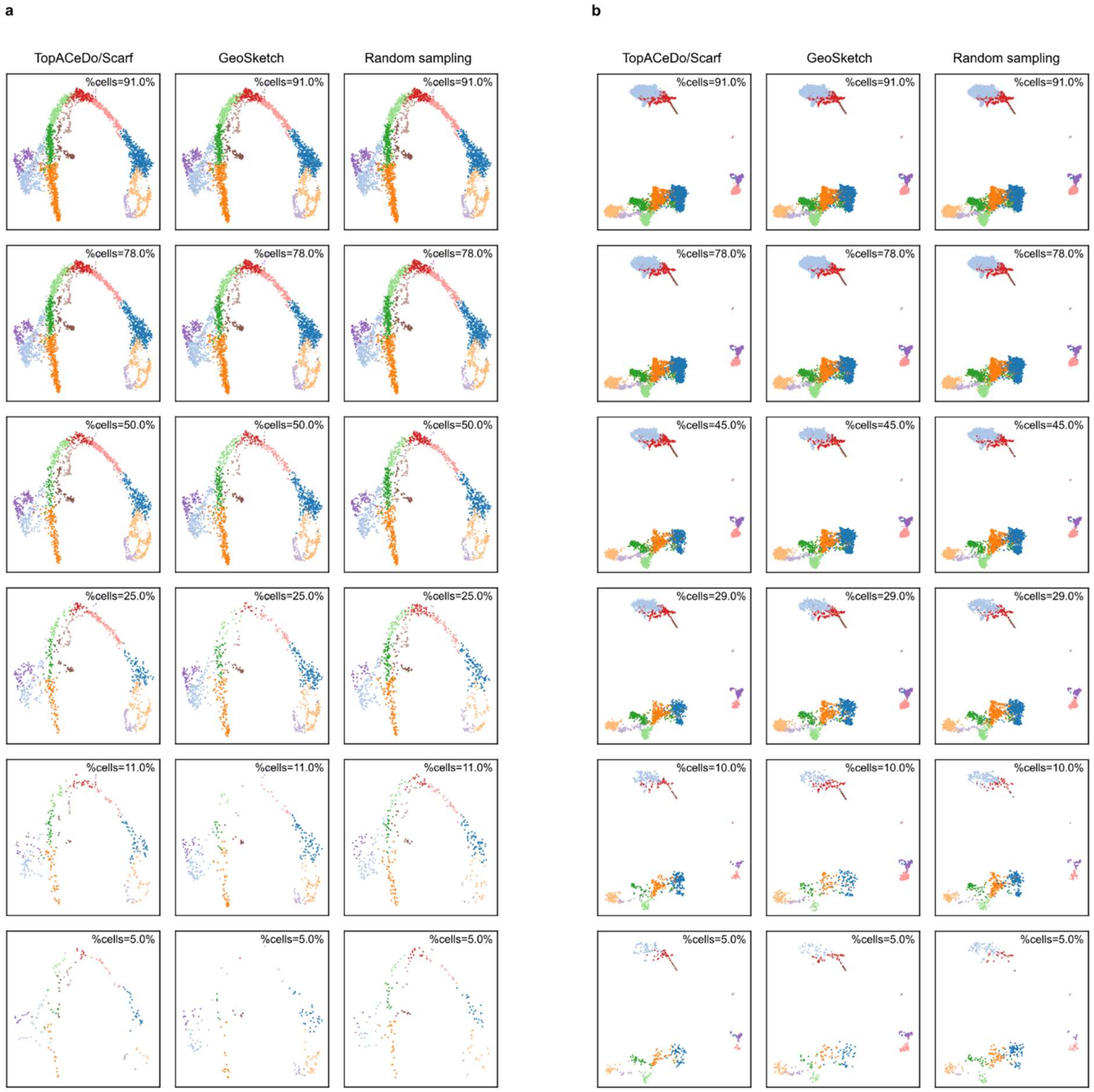

<u>Supplementary Figure 3</u>: Multiple levels of downsampling using TopACeDo algorithm. (a-b) UMAP plots showing downsampled cells after applying TopACeDo, GeoSketch and random sampling to Bastidas et. al. **(a)** and 10K PBMC dataset **(b)**. The plots are arranged to show a progressively increasing degree of downsampling.

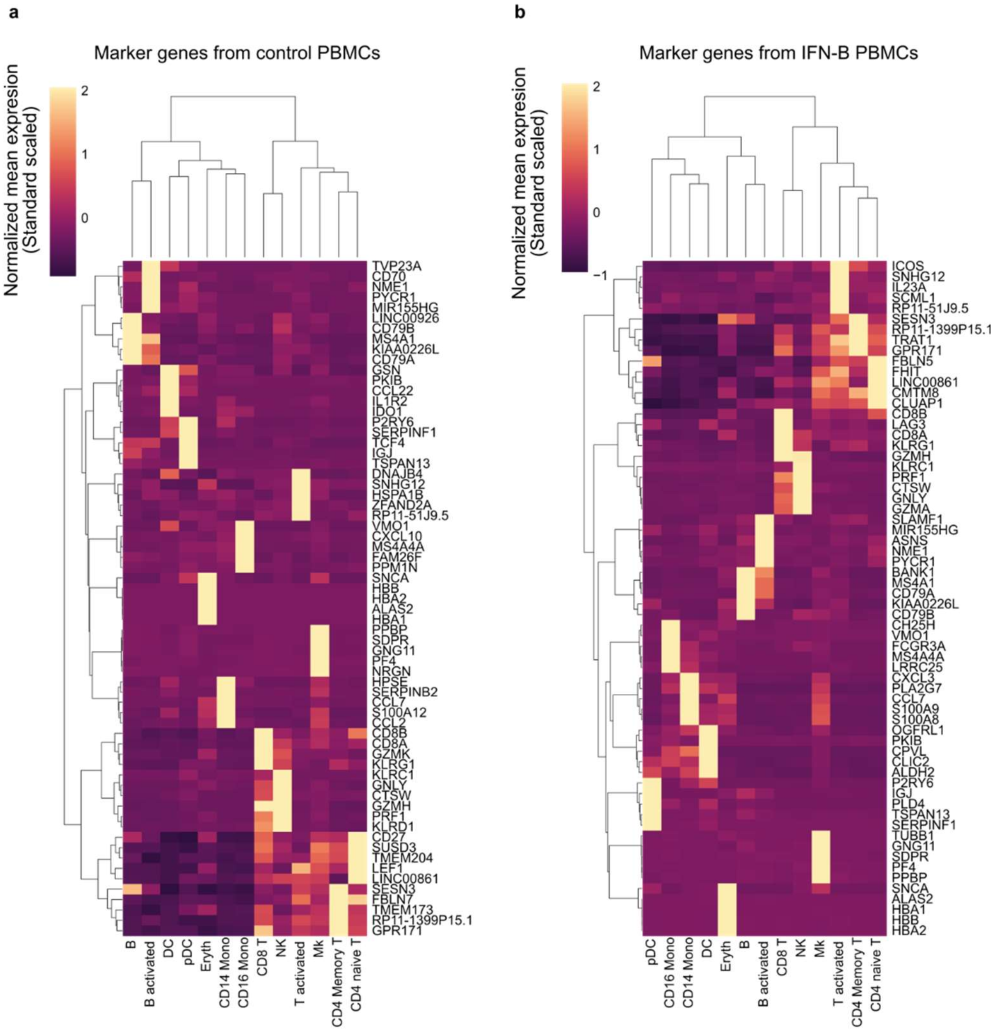

Supplementary Figure 4: Data integration using Scarf. (**a-b**) Heatmap showing z-scaled normalized expression values of top five marker-genes of each cluster from untreated PBMCs **(a)** and IFN-B treated PBMCs **(b)** from Kang et al.

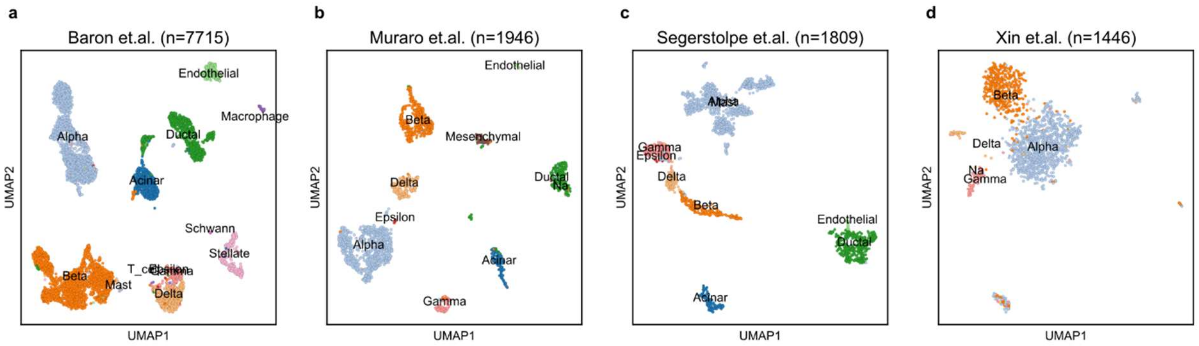

<u>Supplementary Figure 5</u>: Data integration using Scarf. (a-d) UMAP embedding of cells showing original author annotated cell type labels from Baron et al. **(a)** Muraro et. al. **(b)** Segerstolpe et al. **(c)** and Xin et al **(d)**. The numbers in the subplot titles indicate the number of cells in the embedding.

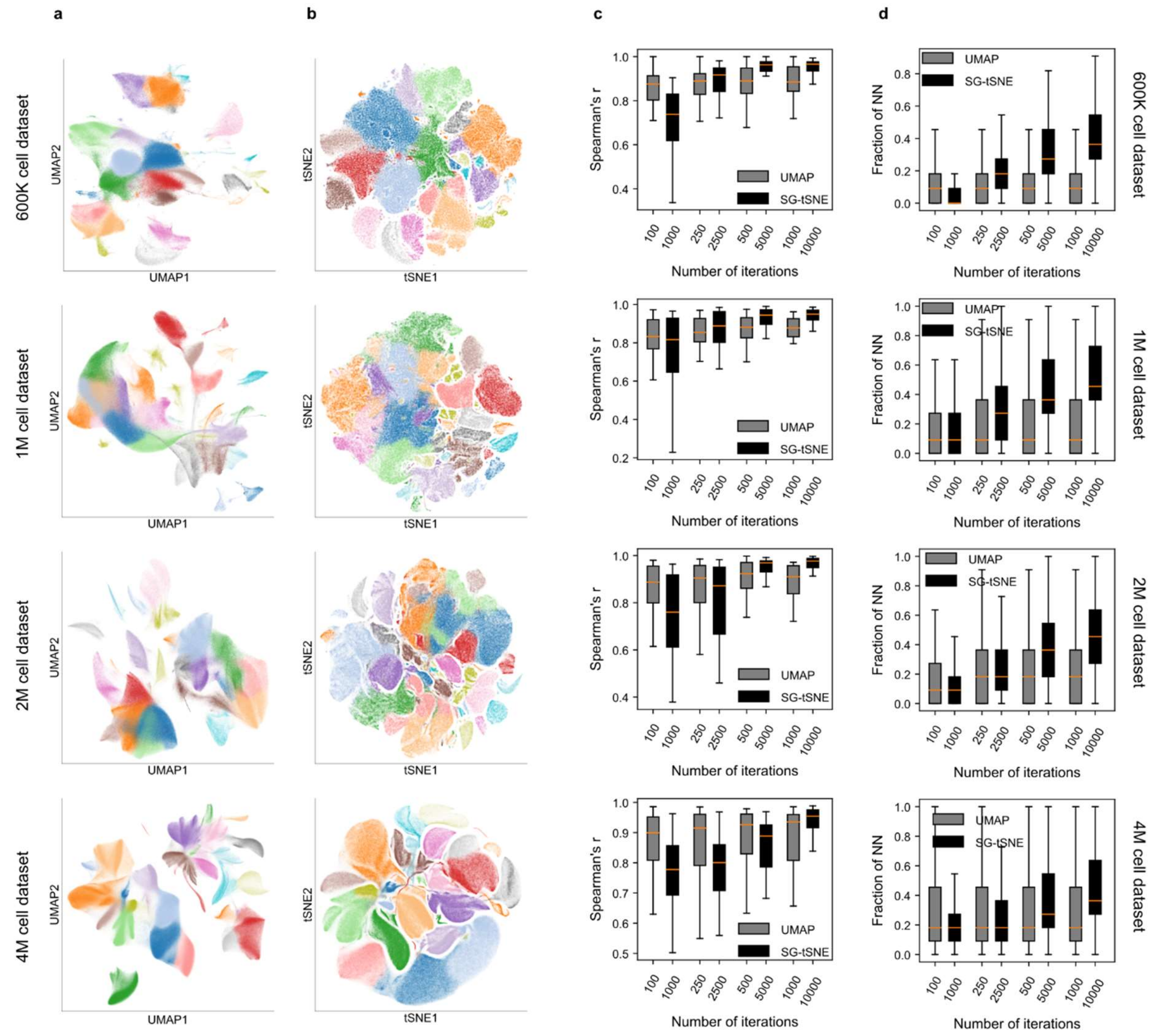

Supplementary Figure 6: Comparison between UMAP and SG-tSNE. (a) Plots showing UMAP embedding (250 iterations) of atlas scale datasets. **(b)** Plots showing SG-tSNE embedding (2500 iterations) of atlas scale datasets. For both UMAP and SG-tSNE, cells are coloured based on Leiden cluster labels. The plots show the density of cells at each pixel to prevent overplotting of cells. **(c)** Boxplots showing the distribution of Spearman's coefficient values calculated between similarity matrices (cluster by cluster) of cell-cell neighbourhood graph and shown embeddings, across four atlas scale datasets. The x-axis shows the progressively increasing number of iterations of UMAP and SG-tSNE that were used to generate the embedding. Each box plot is composed of datapoints representing each cell in the corresponding sample; hence n=728885 (600K dataset), n=1162572 (1M dataset), n=1819817 (2M dataset), n=4764942 (4M dataset). **(d)** Boxplots showing the distribution of the fraction of nearest neighbours that were conserved in the labelled embeddings, across four atlas scale datasets. Each box plot is composed of datapoints representing number of Leiden clusters in the dataset; hence n=26 (600K dataset), n=30 (1M dataset), n=24 (2M dataset), n=30 (4M dataset). Boxplot (c-d) description: the median values are marked using red lines in the boxes, the boxes represent the data between the first and the third quartile, the bottom whisker indicate the values from minimum (after removing outliers) to first quartile and the top whisker indicates from third quartile to adjusted maximum (after removing outliers).

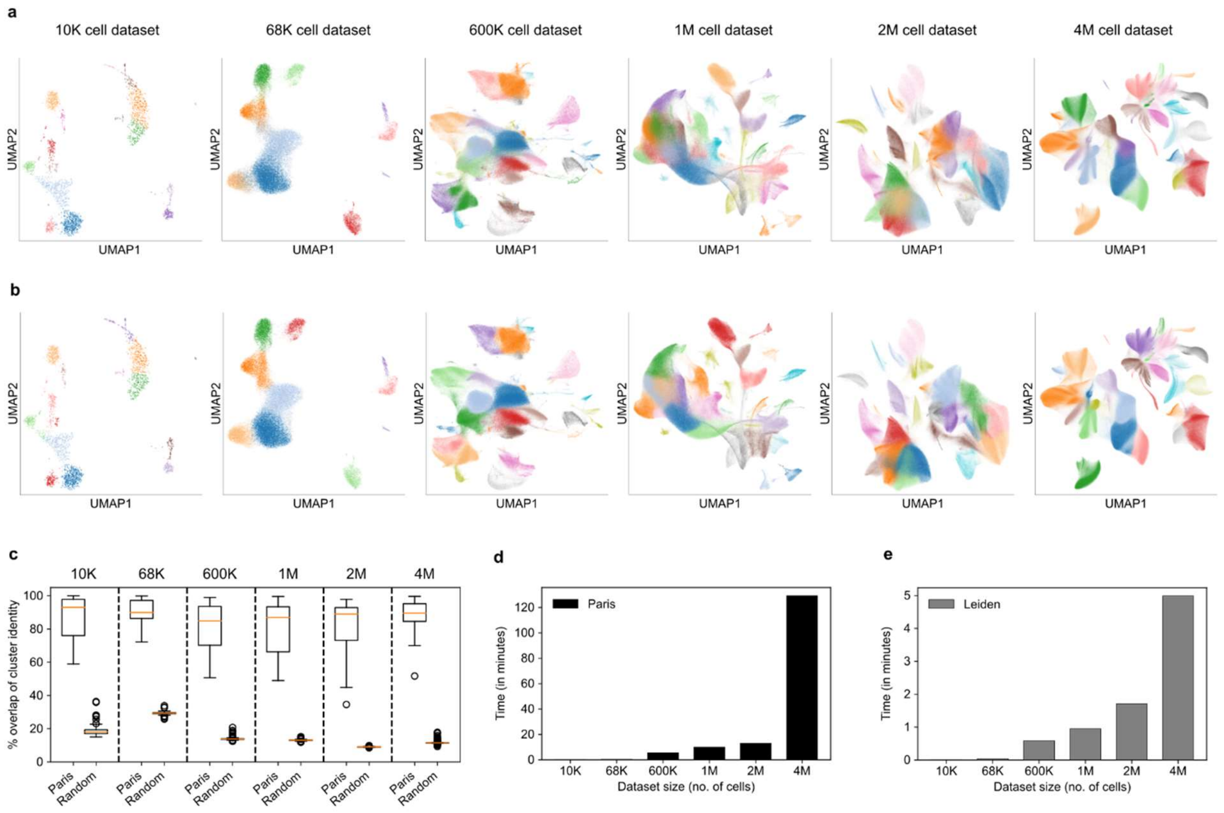

<u>Supplementary Figure 7</u>: Comparison between Paris and Leiden clustering algorithms. (a-b) Plots showing UMAP embedding of cells, coloured according to cluster identity obtained using Paris **(a)** and Leiden **(b)** clustering algorithm, across six datasets. **(c)** Boxplots showing the percentage overlap between cluster identity of cell between either Leiden and Paris or Leiden and random clustering. Each box plot when comparing to Paris clustering is composed of datapoints representing number of Leiden clusters in the dataset; hence n=15 (10K dataset), n=10 (68K dataset), n=26 (600K dataset), n=30 (1M dataset), n=24 (2M dataset), n=30 (4M dataset). When comparing to random clustering, each boxplot of is composed of 10 times (due to 10 random iterations) the numbers indicated above for each dataset. Boxplot description: the median values are marked using red lines in the boxes, the boxes represent the data between the first and the third quartile, the bottom whisker indicate the values from minimum (after removing outliers) to first quartile and the top whisker indicates from third quartile to adjusted maximum (after removing outliers). The outliers (identified using 1.5 IQR rule, as implemented in Matplotlib library) are shown as circles. The approximate number of cells in the datasets are indicated on top of each subplot. **(d-e)** Bar plots showing the time consumption (in minutes) of Paris **(d)** and Leiden **(e)** clustering on the six benchmarked datasets.

		Labels transferred from control PBMCs												
		B	B activated	CD 14 Mono	CD16 Mono	CD4 Memory T	CD4 naive T	CD8 T	DC	Eryth	Mk	NK	T activated	pDC
**Annotated cell types of IFN-B treated PBMCs**	B	**95.5**	14.7	0.5	0	1.4	0.5	0.6	0	0	1.2	9.4	1.2	1.4
	B activated	1.1	**83.7**	0	0	0.1	0	0	0	0	0	0	0	0
	CD 14 Mono	0.1	0	**89.1**	0.4	0.3	0.1	0	7.8	0	1.2	0	0	0
	CD16 Mono	0	0	4.7	**99.6**	0	0	0	0	0	3.1	0	0	0
	CD4 Memory T	0.4	0	0.5	0	**74.3**	11	6.8	0	0	1.2	0.2	7.8	0
	CD4 naive T	2	1.1	1.1	0	14.9	**85.5**	1.7	0	0	3.7	0.2	9	1.4
	CD8 T	0	0	0.4	0	6.5	0.5	**82.5**	0	0	0.6	8.8	0.6	0
	DC	0	0.5	2	0	0.1	0	0	**90.4**	0	0	0	0	4.1
	Eryth	0.1	0	0	0	0.1	0	0	0	**97.6**	0	0	0	1.4
	Mk	0.1	0	1.2	0	0.4	0.8	0.5	0.6	0	**89**	0	0.6	0
	NK	0	0	0.4	0	0.1	0	7.3	0	0	0	**81.4**	0	0
	T activated	0.6	0	0.1	0	1.6	1.5	0.5	0	2.4	0	0	**80.7**	0
	pDC	0	0	0	0	0	0	0	1.2	0	0	0	0	**91.9**

Supplementary Table 1: **KNN projection-based label transfer from control PBMCs to IFN-B treated PBMCs**. The values represent the percentage of predicted/transferred labels against their author annotated cell type.

		Labels transferred from Baron et. al.												
		ACINAR	ALPHA	BETA	DELTA	DUCTAL	ENDOTHELIAL	EPSILON	GAMMA	MACROPHAGE	MAST	NA	SCHWANN	STELLATE
**Muraro et al. cell types**	ACINAR	**92.9**	0.1	1.7	0	12	0	0	0	0	0	0	0	0
	ALPHA	0	**98.8**	6	1.3	1.7	0	0	20.8	0	0	53.8	0	0
	BETA	3.1	0.4	**81.8**	0.7	1.2	0	0	2.1	8.3	0	15.4	0	1.4
	DELTA	0	0.1	9.5	**75.8**	0	0	0	25	0	0	15.4	0	0
	DUCTAL	1	0	0.2	0	**83.4**	0	0	0	91.7	100	0	100	1.4
	ENDOTHELIAL	0	0	0	0	0	**100**	0	0	0	0	0	0	1.4
	EPSILON	0	0.1	0	0	0	0	**100**	0	0	0	3.8	0	0
	GAMMA	3.1	0.4	0.8	22.2	0	0	0	**52.1**	0	0	11.5	0	0
	MESENCHYMAL	0	0	0	0	0.4	0	0	0	0	0	0	0	95.8

Supplementary Table 2: **KNN projection-based label transfer of pancreatic cells from Baron et. al to pancreatic cells from Muraro et. al**. The values represent the percentage of predicted/transferred labels against their author annotated cell type.

		Labels transferred from Baron et. al.										
		ACINAR	ALPHA	BETA	DELTA	DUCTAL	ENDOTHELIAL	GAMMA	MACROPHAGE	MAST	NA	STELLATE
**Segerstolpe et al. cell types**	ACINAR	**100**	0.1	8.2	0	4.5	0	0	0	0	0	0
	ALPHA	0	**99.6**	21.7	1.4	0	0	29.2	100	0	78.6	23.1
	BETA	0	0	**66.9**	0	0	0	0	0	0	0	0
	DELTA	0	0	0.4	**42.3**	0	0	0	0	0	0	0
	DUCTAL	0	0.3	0.4	0	**95.5**	0	0	0	0	7.1	53.8
	ENDOTHELIAL	0	0	0	0	0	**100**	0	0	0	0	0
	EPSILON	0	0	0	0.5	0	0	6.2	0	0	7.1	7.7
	GAMMA	0	0	2.5	55.8	0	0	**64.6**	0	0	7.1	15.4
	MAST	0	0	0	0	0	0	0	0	100	0	0

Supplementary Table 3: **KNN projection-based label transfer of pancreatic cells from Baron et. al to pancreatic cells from Segerstolpe et. al**. The values represent the percentage of predicted/transferred labels against their author annotated cell type.

		Labels transferred from Baron et. al.												
		ACINAR	ALPHA	BETA	DELTA	DUCTAL	ENDOTHELIAL	EPSILON	GAMMA	MACROPHAGE	MAST	NA	SCHWANN	STELLATE
**Xin et al. cell types**	ALPHA	66.7	**99.6**	7.8	9.7	57.1	63.6	100	77.6	50	33.3	81	100	68
	BETA	13.3	0.3	**92.2**	1.1	30.2	27.3	0	2	50	66.7	12.7	0	12
	DELTA	13.3	0.1	0	**34.4**	4.8	9.1	0	0	0	0	2.5	0	12
	GAMMA	6.7	0	0	53.8	7.9	0	0	**20.4**	0	0	3.8	0	8

Supplementary Table 4: **KNN projection-based label transfer of pancreatic cells from Baron et. al to pancreatic cells from Xin et. al**. The values represent the percentage of predicted/transferred labels against their author annotated cell type.
