# Supplementary material for: Scarf enables a highly memory-efficient analysis of large-scale single-cell genomics data
Source: Nat Commun. 2022 Aug 8;13:4616. doi: 10.1038/s41467-022-32097-3 (PMC9360040; doi:10.1038/s41467-022-32097-3)
Supplement: Supplementary file 2 — Reporting Summary [file 41467_2022_32097_MOESM2_ESM.pdf]

nature research

Corresponding author(s): Göran Karlsson
Parashar Dhapola

Last updated by author(s): May 6, 2022

Reporting Summary

Nature Research wishes to improve the reproducibility of the work that we publish. This form provides structure for consistency and transparency in reporting. For further information on Nature Research policies, see our Editorial Policies and the Editorial Policy Checklist.

Statistics

For all statistical analyses, confirm that the following items are present in the figure legend, table legend, main text, or Methods section.

n/a	Confirmed	
☐	☒	The exact sample size (*n*) for each experimental group/condition, given as a discrete number and unit of measurement
☒	☐	A statement on whether measurements were taken from distinct samples or whether the same sample was measured repeatedly
☐	☒	The statistical test(s) used AND whether they are one- or two-sided<br>*Only common tests should be described solely by name; describe more complex techniques in the Methods section.*
☒	☐	A description of all covariates tested
☒	☐	A description of any assumptions or corrections, such as tests of normality and adjustment for multiple comparisons
☐	☒	A full description of the statistical parameters including central tendency (e.g. means) or other basic estimates (e.g. regression coefficient) AND variation (e.g. standard deviation) or associated estimates of uncertainty (e.g. confidence intervals)
☐	☒	For null hypothesis testing, the test statistic (e.g. *F*, *t*, *r*) with confidence intervals, effect sizes, degrees of freedom and *P* value noted<br>*Give P values as exact values whenever suitable.*
☒	☐	For Bayesian analysis, information on the choice of priors and Markov chain Monte Carlo settings
☒	☐	For hierarchical and complex designs, identification of the appropriate level for tests and full reporting of outcomes
☐	☒	Estimates of effect sizes (e.g. Cohen's *d*, Pearson's *r*), indicating how they were calculated

Our web collection on statistics for biologists contains articles on many of the points above.

Software and code

Policy information about availability of computer code

Data collection	Data was downloaded using wget (1.20.3) Linux utility directly from the sources mentioned below. Anndata2ri library (v.1.0.5) was used to convert RDS objects to Anndata objects.
Data analysis	Scarf (version 0.7.7), Scanpy (version 1.6.1) and Geosketch (version 1.2) were used.<br>Scarf's has following dependencies (version number indicated for Scarf version 0.7.7):<br>Dask (2021.5.1)<br>Zarr (2.8.1)<br>Blosc (1.10.2)<br>Statsmodels (0.11.1)<br>HNSWlib (0.5.1)<br>Gensim (4.0.1)<br>PCST (https://github.com/fraenkel-lab/pcst_fast)<br>scikit-learn (0.24.2)<br>scikit-network (0.23.1)<br>leidenalg (0.8.4)<br><br>full list of Scarf's dependencies can be found here: https://github.com/parashardhapola/scarf/blob/master/requirements.txt<br><br>The source code for Scarf package is available here: github.com/parashardhapola/scarf<br>The notebooks, data and scripts used in the paper can be found here: https://osf.io/cbu6a<br>The documentation for installation and usage of Scarf can be found here: scarf.readthedocs.io.<br>All count matrices used in this study can be obtained using following command:

`scarf.fetch_dataset(dataset_id)`
Ids for all available datasets can be obtained using this command: `scarf.show_available_datasets()`.

For manuscripts utilizing custom algorithms or software that are central to the research but not yet described in published literature, software must be made available to editors and reviewers. We strongly encourage code deposition in a community repository (e.g. GitHub). See the Nature Research guidelines for submitting code & software for further information.

Data

Policy information about availability of data

All manuscripts must include a data availability statement. This statement should provide the following information, where applicable:

- Accession codes, unique identifiers, or web links for publicly available datasets
- A list of figures that have associated raw data
- A description of any restrictions on data availability

- Kang et. al [28] kang_15K_pbmc_rnaseq : GSM2560248 (GEO)
- Kang et. al [28] kang_14K_ifnb-pbmc_rnaseq : GSM2560249 (GEO)
- Baron et. al [30] baron_8K_pancreas_rnaseq: GSE84133(GEO)
- Muraro et. al [31] muraro_2K_pancreas_rnaseq : GSE85241 (GEO)
- Segerstolpe et. al [32] segerstolpe_2K_pancreas_rnaseq: E-MTAB-5061 (ArrayExpress)
- Xin et. al [33] xin_1K_pancreas_rnaseq : GSE81608 (GEO)
- Zeisel et. al [34] zeisel_161K_nervous_rnaseq: https://storage.googleapis.com/linnarsson-lab-loom/l5_all.loom
- Saunders et. al [59] saunders_110K_brain_rnaseq: http://dropviz.org/
- 10x genomics Data datasets [] tenx_8K_pbmc_citeseq: http://cf.10xgenomics.com/samples/cell-exp/3.0.0/pbmc_10k_protein_v3/pbmc_10k_protein_v3_filtered_feature_bc_matrix.h5
- Bastidas-Ponce et. al [60] bastidas-ponce_4K_pancreas-d15_rnaseq: https://github.com/theislab/scvelo_notebooks/raw/master/data/Pancreas/endocrinogenesis_day15.h5ad
- Zheng et. al [16] zheng_69K_pbmc_rnaseq: http://cf.10xgenomics.com/samples/cell-exp/1.1.0/fresh_68k_pbmc_donor_a/fresh_68k_pbmc_donor_a_filtered_gene_bc_matrices.tar.gz
- Human Cell Atlas Data Portal [] hca_783K_blood_rnaseq: https://data.humancellatlas.org/project-assets/project-matrices/cc95ff89-2e68-4a08-a234-480eca21ce79.homo_sapiens.mtx.zip
- 10x genomics datasets [] tenx_1.3M_brain_rnaseq: http://cf.10xgenomics.com/samples/cell-exp/1.3.0/1M_neurons/1M_neurons_filtered_gene_bc_matrices_h5.h5
- Cao et. al [17] cao_2.1M_moca_rnaseq : GSE119945 (GEO) https://shendure-web.gs.washington.edu/content/members/cao1025/public/mouse_embryo_atlas/gene_count.txt
- Cao et. al [18] cao_4.9M_fetal_rnaseq: GSE156793 (GEO) https://descartes.brotmanbaty.org/bbi/human-gene-expression-during-development/
- Domcke et. al [19] domcke_721K_fetal_atacseq : GSE149683 (GEO) https://descartes.brotmanbaty.org/bbi/human-chromatin-during-development/

All count matrices used in this study can be obtained using the following command:
scarf.fetch_dataset(dataset_id)

Ids for all available datasets can be obtained using this command:
scarf.show_available_datasets()

Field-specific reporting

Please select the one below that is the best fit for your research. If you are not sure, read the appropriate sections before making your selection.

☒ Life sciences ☐ Behavioural & social sciences ☐ Ecological, evolutionary & environmental sciences

For a reference copy of the document with all sections, see nature.com/documents/nr-reporting-summary-flat.pdf

Life sciences study design

All studies must disclose on these points even when the disclosure is negative.

Sample size	Publicly available datasets were used for this study. The number of datasets were chosen based on the size of the datasets. For example, we chose datasets with approx, 10K, 60K, 500K, 1M, 2M and 4M cells to adequately test the scalability of different software
Data exclusions	Cells from single-cell datasets that did not meet the quality thresholds of number of reads, number of genes, % mitochondrial and % ribosomal contamination were removed. Such exclusion were part of data analysis pipeline. Otherwise no data was excluded from the study
Replication	To adequately test that the runtime and memory utilization of software were not affected by server load from other users, we ran three instances (technical replicated) of analysis pipeline for each of the benchmarked software. We observed only very minor differences as the differences between the runs were an order of magnitudes smaller than runtime. Same was true for memory consumption.
Randomization	We did not perform our analysis from any cohort, but rather used published datasets in their entirety

Blinding	We used previously published data and did not perform any test-control experiments in this study that necessitated blinding.

Reporting for specific materials, systems and methods

We require information from authors about some types of materials, experimental systems and methods used in many studies. Here, indicate whether each material, system or method listed is relevant to your study. If you are not sure if a list item applies to your research, read the appropriate section before selecting a response.

Materials & experimental systems

n/a	Involved in the study
☒	☐ Antibodies
☒	☐ Eukaryotic cell lines
☒	☐ Palaeontology and archaeology
☒	☐ Animals and other organisms
☒	☐ Human research participants
☒	☐ Clinical data
☒	☐ Dual use research of concern

Methods

n/a	Involved in the study
☒	☐ ChIP-seq
☒	☐ Flow cytometry
☒	☐ MRI-based neuroimaging
